# Networking chemical robots for reaction multitasking

**DOI:** 10.1038/s41467-018-05828-8

**Published:** 2018-08-24

**Authors:** Dario Caramelli, Daniel Salley, Alon Henson, Gerardo Aragon Camarasa, Salah Sharabi, Graham Keenan, Leroy Cronin

**Affiliations:** School of Chemistry, The University of Glasgow, Glasgow, G12 8QQ UK

## Abstract

The development of the internet of things has led to an explosion in the number of networked devices capable of control and computing. However, whilst common place in remote sensing, these approaches have not impacted chemistry due to difficulty in developing systems flexible enough for experimental data collection. Herein we present a simple and affordable (<$500) chemistry capable robot built with a standard set of hardware and software protocols that can be networked to coordinate many chemical experiments in real time. We demonstrate how multiple processes can be done with two internet-connected robots collaboratively, exploring a set of azo-coupling reactions in a fraction of time needed for a single robot, as well as encoding and decoding information into a network of oscillating reactions. The system can also be used to assess the reproducibility of chemical reactions and discover new reaction outcomes using game playing to explore a chemical space.

## Abstract (web summary)

Distributing a reaction workload across laboratories can solve chemical problems more efficiently, but it is challenging to develop viable hardware and software. Here, the authors present an internet-connected network of cheap robots that can perform chemical reactions and share outcomes in real time, demonstrating a digitized approach to chemical collaboration.

## Introduction

**Fig. 1:**
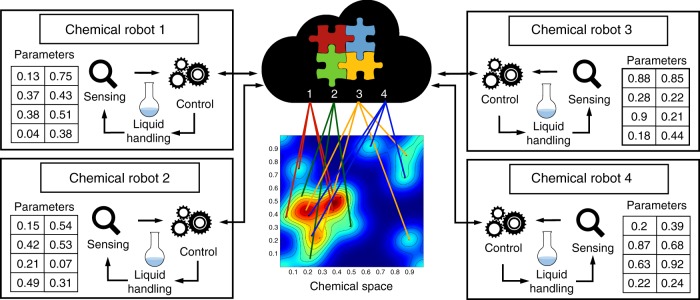
Schematic describing the concept of real-time networked chemical robots. Here four physically separated units (ChemPUs) are connected to a cloud via the internet. They receive the reactions parameters from the cloud in order to explore a chemical space in an optimised way, when the reactions are done the analysis results are returned and shared trough the cloud

The digitisation of everyday life through the computer and internet revolution^1^ has led to systems that allow error-correction, distributed ‘multi-core’ working^2^ and gamification of task-based work^3,4^. Thus, digitisation has led to an explosion in cooperativity driven by common standards and protocols, which means that tasks centrally managed can be distributed over many sites, yet this approach has yet to impact the field of chemistry^4,5^. However, the automation of chemical reactions has been an expanding field in the last decade, including flow chemistry, peptide and nucleic acid synthesis^6^. These robotic systems are specialised and expensive ($50–500K) so the adoption of such automation in chemistry has been limited. This is because using robots to do chemistry is hard due to the bespoke nature of many chemical operations reactions resulting from a lack of standards^7,8^. We hypothesised that the connection of robots capable of doing chemical reactions in real time by the internet could lead to new approaches to explore many chemical reactions, assess reproducibility and control complex chemical reactions in real time. Conceptually, we started by imagining a chemical cloud whereby the code to control a number of identical robots could be held remotely or distributed all the robots. By sharing a common hardware, list of chemical reactions and code the robots could collaborate by doing common chemical tasks simultaneously but decentralised over several laboratories in different locations (Fig. 1).

Similar to the world of distributed and cloud computing our concept can act as a cluster system to explore large chemical spaces^9^ by distributing the workload across the robots connected to the network. Our aim is that the results can be easily reproduced anywhere and anytime by a similar machine, increasing the reliability of chemical research allowing validation of new procedures and discoveries^10^.

## Results

### Platform

To investigate this concept, we designed a system around a low power single board pcDuino3 computer running the Linux Ubuntu operating system (Supplementary Methods 1). We then built a software platform, using Python, to control the robot and the sensor system. The liquid handling system comprises a number of peristaltic pumps connected to a driver board and the sensor array is a single webcam connected to the pcDuino3 via USB. Both the hardware and the software are modular and can be easily upgraded to include other chemical effectors and sensors. By using a common software base, it is possible to tailor the system for a particular chemical problem combining the three main parts of the robot with related external libraries. Communication is achieved via a network (Wi-Fi or ethernet) connection so the robot can broadcast its state and communicate with the other units. As a proof of concept all communication has been conducted through the Twitter platform or by a bespoken server system. The data collected are analysed locally on each board and sent as plain and readable text to the Twitter account or server. In this way the posts are then acquired and can be processed by any other board on the network. For the work presented in this manuscript a total of six identical robots have been built. The reactions are performed in a flask with magnetic stirring, each is washed and reused for each experiment.

### Agent-based model simulation

**Fig. 2:**
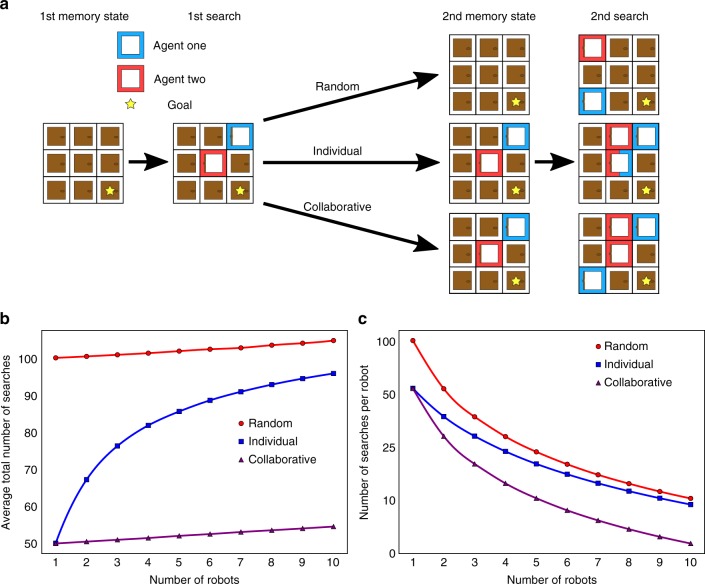
Agent-based simulations. **a** Scheme of the simulated search with the three different strategies. **b** Average number of searches needed under each strategy as a function of the number of robots. **c** Search efficiency in terms of the total number of searches that are needed on average

The principle of multi-threaded networking^11^ of individual chemical robots can be showed by considering simulations of the relevant strategies using an agent-based modelling approach (Fig. 2 and Supplementary Method 5). Here each agent represents a chemical robot as an entity that does all the possible reactions until the goal is achieved. Different strategies can be used by the agents: in the random strategy, the most basic, each robot has no memory of the reactions attempted. In contrast an approach where the robot has memory of the previous reactions increases the probability of finding the goal in the next move (individual approach), and when the number of robots is increased, and they can see the history of all the previous moves, all the robots can work together collaboratively solving the problem more quickly (Fig. 2a). This shows that as the number of robots increases the number of experiments each robot has to do decreases with the best efficiency when the collaborative approach is adopted. Figure 2b shows how the collaborative approach allows the system to maintain a low average number of searches as the amount of robots increase, while the individual approach tends to be inefficient due to the higher chances of repeating experiments already performed by another unit (Fig. 2c).

### Azo-dye collaborative chemical space exploration

**Fig. 3:**
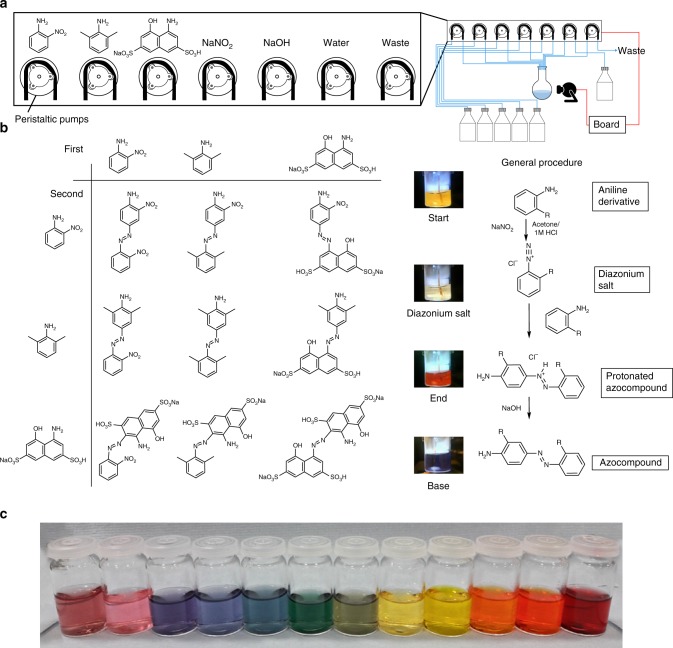
Organic azo-dye chemical space. **a** System connections and reagents assignment to the pumps. **b** Three aniline derivatives were mixed in different order with sodium nitrite in an azo-coupling reaction (general procedure on right-hand side). The starting materials were chosen purposely to be used both as first and second reagents. **c** The addition of a basic solution to the product and a set of reagent ratios lead to a large variety of colours

For many chemical searchers, the brute force exploration of many different combinations of reagents and concentrations is a powerful way to optimise a reaction, or to discover new reactivity. In recent years most of this workload can be automated in the laboratory, but the individual worksites are isolated from each other. We envisaged that by networking two or more robots capable of doing a given set of reactions, it would allow the robots to collaborate with each other, even if they are physically separated over a range of different sites anywhere in the world. To realise this practically in the laboratory we deployed our robots to explore a reaction grid forming a range of dye colourants looking for a set of specific colours^12^. They did this by mixing two different reagents together in a clean sample vial and the results of each reaction were automatically recorded with a webcam (Fig. 3a), analysed and shared in real time using Twitter. By reading each other’s Twitter feed the robots were able to collaboratively search the space and reduce the total number of experiments required to reach the goal of exploring colour space (Supplementary Method 2.1).

**Fig. 4:**
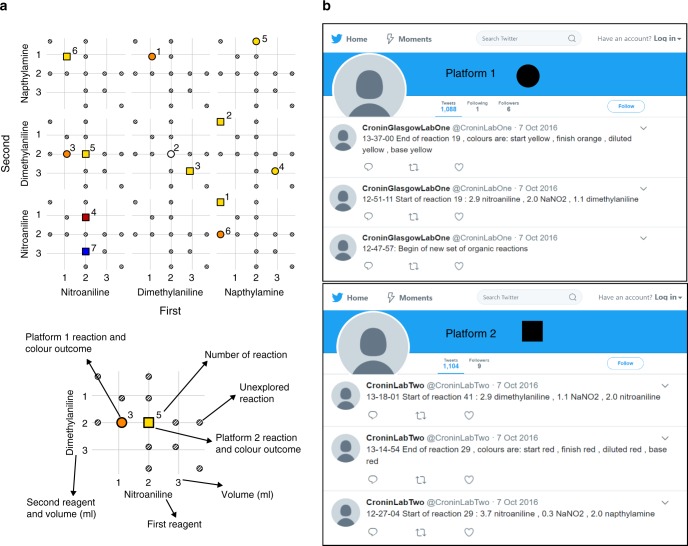
Platform collaboration. **a** Example of the organic space exploration managed by two collaborating units (squares and circles). **b** Twitter accounts used for real-time data sharing

During the chosen reaction two aniline derivatives are mixed with sodium nitrate in an azo-coupling reaction. After ca. 30 min the synthesis of the azo compound can be confirmed by a colour change and the colour of the solution is recorded and analysed in real time (Supplementary Method 2.2). To cover the largest number of distinct colours using the least number of starting materials three aniline derivatives were selected: *o*-nitro-aniline; 2-6-dimethyl-aniline; and sodium 4-amino-5-hydroxy-2,7-napthalenedisulfonate hydrate. Each starting material could be used both as a first component (to synthesise the diazonium salt with the amine group) or as the second component (for the substitution on the benzene ring). The nine molecules obtained from the grid (Fig. 3b) were expanded further by using 13 different ratios for each reaction, obtaining 117 possible combinations. Due to the chemistry involved some of the molecules synthesised acted as pH indicators, therefore a fixed amount of base was added after each reaction to check for a colour change. Overall, the time for each reaction, including cleaning and dispensing was around 40 min, and during each experimental cycle the algorithm was designed to select a random reaction and share the chosen parameters via Twitter or the server. Next, the system then performs the selected reaction and saves four representative images of the reaction, which are analysed locally and shared online (Fig. 4).

A parallel background process checks the other robots every 5 min for Tweets and updates the database with the results respectively. This allows both boards to have the same data stored in their internal databases, avoiding doing the same reaction twice. The search has been successfully run several times, and this shows that the average number of reactions to find the rare blue-coloured one is halved. Also, during the exploration of the full grid, some unexpected colours were observed, e.g., green and pink, which are rare to associate with azo-dye motifs (Fig. 3c).

### Real-time control of oscillating reaction

To explore the real-time aspect of the networked chemical robots, we investigated a chemical oscillator based on the Belousov–Zhabotinsky (BZ) reaction^13^, whereby in the system two physically separated oscillators have been synchronised in real time^14,15^. The reaction consists of the oxidation of malonic acid by potassium bromate, catalysed by a metal-complex in acidic aqueous solution, oscillations are visible as blue/red colour change thanks to the ferroin that acts both as catalyst and indicator. Initially, the robots begin the reactions at different starting points and, through image analysis, data sharing and chemical adjustments, they can use the synchronisation algorithm to reach identical oscillation periods. By ensuring constant stirring, the oscillations show stable dynamics via the webcam, and the period is recorded and calculated on-board the robot with a real-time image analysis algorithm. Different strategies to achieve a control over the oscillation period have been reported in the literature, either by using the ratio of starting materials, the temperature^16^ or the stirring rate^17^. Here we modulate the oscillation period in real time while the reaction is already running by using small additions of starting materials. To do this, potassium bromate and water were selected respectively to increase and decrease the oscillation period through a series of controlled additions where two functions were used to predict the behaviour of the reaction.

**Fig. 5:**
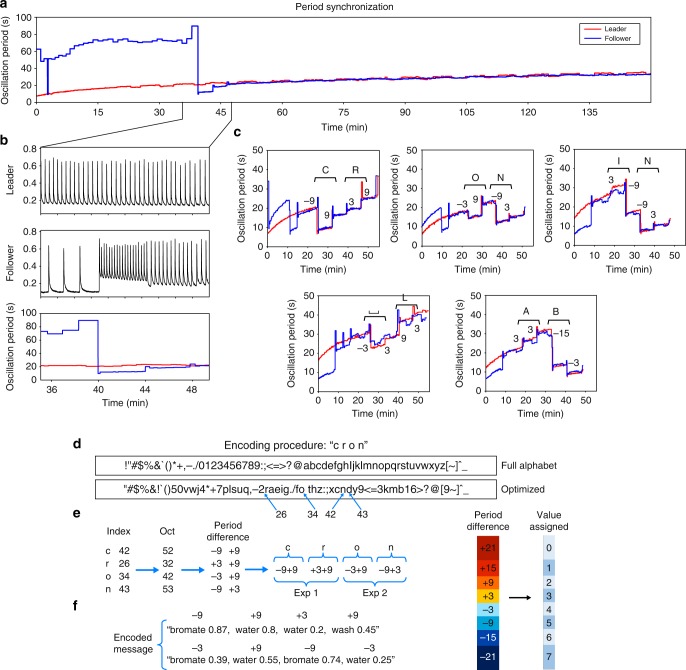
Real-time control of chemical oscillator. **a** BZ reaction synchronisation achieved by two units acting as leader and follower. **b** Zoomed plot of the periods, at 40 min the follower adds potassium bromates and overshoots, the period is then corrected with two small additions of water and is synchronised at 48 min. **c** Real data of encoding/decoding of ‘cronin lab’. Encoding of the word ‘cron’. **d** Each character is converted into the relative number using the optimised alphabet. **e** These numbers are converted first into octal and then into a frequency change by using the threshold table. **f** Final encoded message, each character is represented by two reagent additions. The amounts are reported as an example since real amounts are dynamic and are calculated in real time by monitoring the oscillation frequency

While one board acted as the ‘leader’, simply sharing its period every 4 min, the other acted as a ‘follower’, trying to synchronise its own period with the one of the leader (Fig. 5a, b). Within a few iterations, and by applying the empirical functions, the two periods have successfully been synchronised in real time with an uncertainty of 2 s. To explore this, the periods of both platforms were recorded for 90 min showing that the reactions kept oscillating at the same frequency. To demonstrate the reliability of the platform we managed to send a message between two oscillating systems by encoding it into a change in frequency (Fig. 5c). The message is split into individual characters and each is converted into a number using an optimised alphabet (Fig. 5d). The number is converted into octal numerical system (base-8 numbers), the digits of obtained octal are expressed using the degree of modulation of the reaction frequency by using the threshold table (Fig. 5e). The experiment starts with two separate systems oscillating with different periods. When synchronisation is achieved the leader adds the material to change its frequency by the difference associated with the message, then sends the amount added to the follower (Fig. 5f). The follower adds this amount and measures the value of the new frequency. The new frequency should be the same for both systems and the difference is the encoded message. In Fig. 5 the details of the encoded message for the partial word ‘cron’ is showed as an example, however the amount of material to obtain the period difference depends on the current period of the reaction and is calculated in real time during the encoding/decoding. Therefore, the encoded message is not a direct translation but depends on leader’s reaction period and can be successfully decoded only if the follower’s reaction is oscillating at the same speed. Since a single experiment can hold up to four additions in a reliable way it is possible to send two characters for each BZ reaction before proceeding to the automatic clean cycle. However, a programme to perform a series of reactions and to send a message of any length has been written and used to successfully encode/decode ‘cronin lab’.

### Reproducibility assessment of inorganic cluster crystallisation

**Fig. 6:**
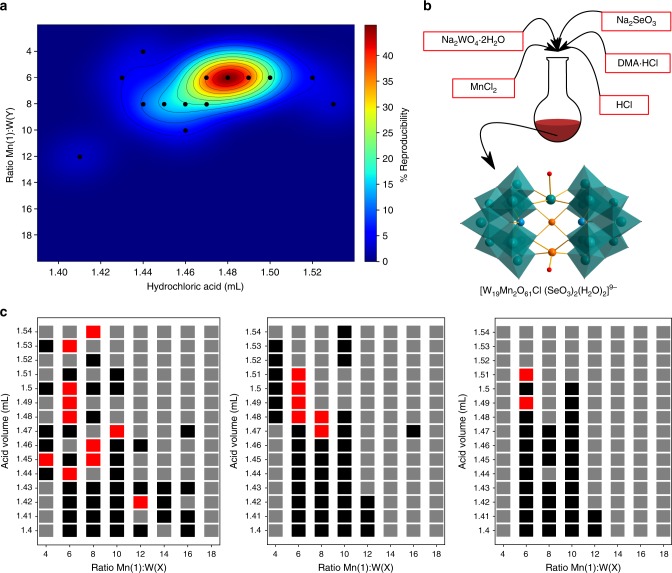
Reproducibility of POM crystallisation. **a** Heat map of % reproducibility of each conditions that produced crystals at least once. **b** One-pot synthesis of the W_19_Mn_2_Se_2_ polyoxometalate cluster with accompanying structure. **c** Three automated grid search results of reaction space (red = crystals, grey = precipitate, black = no crystals)

To collaboratively search chemical space for the evaluation of the reproducibility^18^ of a complex cluster a known tungsten polyoxometalate (POM) cluster^19^ was chosen. First, we set out to establish values of reagent stoichiometry and pH that would produce crystals of the compound within 2 h, monitored by a webcam. The reaction conditions meeting this criterion were repeated to determine the reproducibility of the process. Full automation of the synthesis, reaction recording and crystal recognition were achieved using the platform described above combined with image analysis machine-learning software. A grid of 120 reactions was split into 8 series of 15, each of which was explored collaboratively between two platforms. To start each series, the leader selects at random a reaction from the list of 15 shared between it and the follower and informs the network. The follower repeats this step with the remaining 14 reaction options and the process continues until the series has been completed. In real time the network is informed with feedback about each reaction result, i.e., observing precipitation or the formation of crystals or not. Reagents are added to the reaction vial in sequence and the resulting solution is stirred for 10 min. Following transfer of the reaction solution to a recording vial, suspended over a webcam, the reaction is recorded for 2 h. A machine-learning crystal recognition method was developed by training a model from previous reactions and deployed during the 2-h recording period. Many both clear and crystalized reaction images from previous experiments were compiled into a database to train this model (Methods—Inorganic). The grid of 120 reactions was completed three times between the two collaborating platforms revealing 13 conditions in the space that produced crystals at least once (Fig. 6).

Each of these reaction conditions was repeated until a consistent average percentage of reproducibility emerged. If the reaction reached 15 failed experiments in a row it was abandoned as being too stochastic. Of the 13 reaction conditions to have produced crystals at least once 7 never again produced crystals. The remaining 6 showed percentages of reproducibility between 11.8 and 50% with the optimum reaction conditions being a Mn:W ratio of 1:6 using 1.49 mL of 2.32 M HCl. Given that crystallisation is a stochastic process, collaborative exploration and optimisation to determine both the ideal conditions and likelihood of crystallisation could provide a solution to some aspects of the crisis of reproducibility described in the Introduction.

### Gameplay-driven chemical discovery

**Fig. 7:**
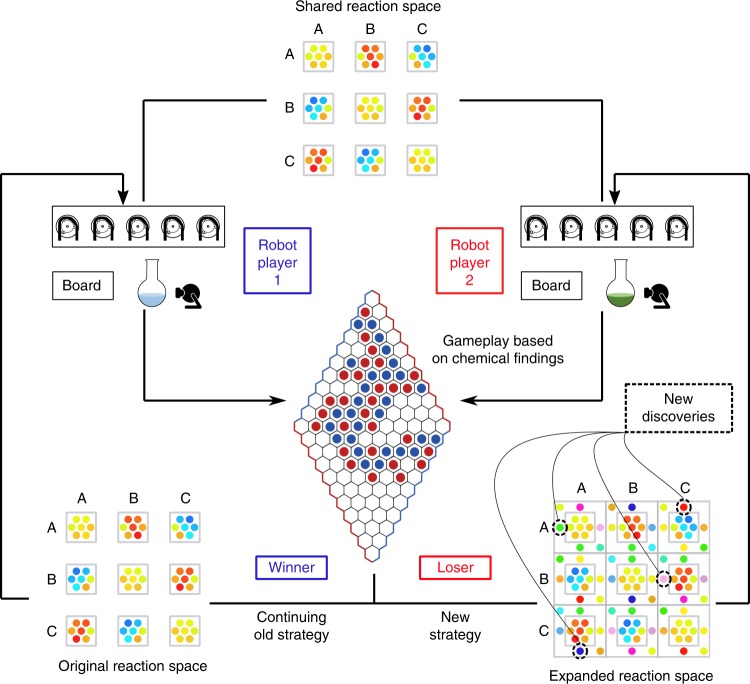
Gameplay algorithm of player 1 vs player 2 in a series of Hex games. After the first game is complete strategies of the winner and loser diverge to explore unknown chemical space

Normally in chemistry, the process to design experiments into a grid is a very well-known and defined process. Using the process of collaboration shown here, we showed how the chemical robots could effectively collaborate by covering more reactions at the same time, by avoiding duplications and sharing the outcomes. We wondered if we could adapt this by allowing the robots to play a game, and then, rather than just doing the reactions as specified, allow them to change the reaction parameters (cleaning, concentration and time) in an effort to develop a strategy to win the game. This is because we linked the outcome of the chemical reactions, in this case which colour the solution changed to, and how often that particular colour was observed, to which move the robot could make on the board game. The game selected for this was Hex^20^, a very simple strategy board game played by two players on a grid of hexagons traditionally shaped as a 11 × 11 rhombus. In the normal game players select a hexagon on the grid and attempt to join one side with the opposite side with a linked set of hexagons (see middle of Fig. 7). In our game the players are each a chemical robot, each with an identical list of chemical reactions that can be done. For the first move, a player randomly selects and performs a reaction from their grid. The chosen reaction space consisted of the same three aniline derivatives described in Fig. 3, and the move each robot can make is determined by the colour of the reaction that each robot performs. The game logic is based on the rarity of the reaction result; if a new/rare colour has been found, the optimal move is allowed, however if the result has been seen many times, a sub-optimal or random move is allowed on the game board. Once the winner of the first game has emerged, the losing robot is allowed to change strategy by changing the variables associated with a given reaction, thereby attempting to find more new/rare colours to allow a better move. In this case the reaction space (possible combinations of the same three aniline compounds) is increased for the loser, in the hope of finding new results (Fig. 7). The platforms communicate via a shared server that updates the live game board, and optimal movements are determined by a Hex game algorithm using Monte-Carlo simulations (Supplementary Method 6).

**Fig. 8:**
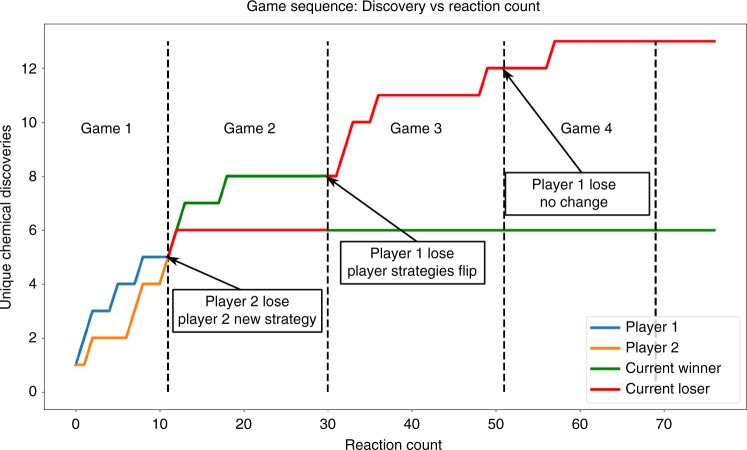
Discoveries over time player 1 vs player 2. A four Hex game sequence between two automated platforms. Game 1: initial game to establish winner/loser (same strategy), game 2: initial loser begins using expanded strategy and wins, no significant change in discoveries, games 3 and 4: players switch strategy for the final time, loser continues to make significantly more discoveries until the series ends

A game sequence can see two to five individual games completed between two players before the reaction space is complete. Figure 8 shows a typical sequence of four complete games (fifth remained incomplete) in which the losing strategy, over time, produces significantly more unique discoveries then a continued search of the original reaction space using the non-gaming approach. By presenting the goal of victory in a game of hex to these platforms and allowing changes to be made in search strategy toward that goal after suffering loss, we have shown the potential for new chemical discovery outside the realm of laborious sequential search methods between networked robotic platforms. This development in the strategy results after feedback from the robot playing the game, the move allowed as a function of the game and then the result of the game allowing the robot to change the strategy to discover new/rare colours.

## Discussion

We have presented a network of robots capable of autonomously performing chemical reactions, analysing them and using the internet to communicate data. The system was designed to be purposely as simple as possible; nevertheless, it was capable to manage three completely different chemical processes, all involving collaborative approaches based on data sharing. A combinatorial grid of organic molecules has been explored in half the time looking for a specific result, showing the advantages of multiple units. By using the real-time communication, the periods of two oscillating reactions have been precisely controlled and used to encode and decode information. Two units performed and reproduced POM syntheses under varied conditions in order to assess the reproducibility percentages of different parameters. Finally, we showed that chemical robots who could only choose the ‘moves’ or reactions to perform as a function of the state of a game are able to make more discoveries compared to the screening approach. We imagine that an expansion in automated systems inside chemistry laboratories, where robots will be used to repeat standard procedures, will allow chemists more time to do more challenging experiments. A network of such robots in distant labs would be easily scalable and might become a worldwide system with thousands of units connected. In terms of security these systems would share an identical communication standard and be protected from unexpected remote intrusions through a username/password login or a VPN tunnel connection in a similar way as online analytical instruments (i.e., nuclear magnetic resonance, high-performance liquid chromatography, mass spectrometry, etc.) are already doing.

## Methods

### Organic

All materials are prepared in stock solutions and stored for several days without any noticeable effect: *o*-nitro-aniline [0.01 M in acetone/HCl 1.7 M 1:1]; 2-6-dimethyl-aniline [0.01 M in acetone/HCl 1.7 M 1:1]; Na 4-amino-5-hydroxy-2,7-napthalenedisulfonate hydrate [0.01 M in acetone/HCl 1.7 M 1:1]; sodium nitrate [0.01 M in water]; and sodium hydroxide [0.5 M in water]. During each reaction the first selected aniline derivative is mixed with sodium nitrate to form the relative diazonium salt. After 2 min the second aniline derivative is added to the solution. When performing the reactions on the bench they showed a colour change after a period between 1 and 60 min, indicating the synthesis of the azo compound. In the platforms a time of 30 min has been used since the majority of the combinations showed complete conversion within this timeframe. The reaction is then diluted by removing 5 mL out of 6 and replacing them with water, then 2 mL of sodium hydroxide solution are added. The reaction is an electrophilic aromatic substitution reaction where the aryldiazonium cation is the electrophile and the benzene ring of the second aniline is the nucleophile. From the initial 3 × 3 grid (Fig. 3b) each reaction has been further expanded using different reagents ratios. Five possible volume ratios (0.3, 1.1, 2.0, 2.9, and 3.7 mL) have been chosen and combined maintaining total volume of 6 mL, obtaining 13 combinations of volumes for the 3 reaction components (Supplementary Fig. 6). The resulting 117 reaction grid has been fully explored with two platforms in 88 h using a simple programme. The board starts by adding the first aniline derivate and sodium nitrate in the flask. After waiting for 2 min for the diazonium salt synthesis it adds the second aniline and waits for 30 min. When the reaction is completed the solution is diluted and the base is added. During each reaction the board records four frames: right after reagents mixing (start); after 30 min (finish); after dilution (diluted); and after the sodium hydroxide solution is added (base). Image frames of the full grid have been analysed with the colour detection algorithm and plotted in order to visualise the colour distribution (Supplementary Fig. 7).

### Physical

Potassium bromate stock solution is prepared fresh every day since it has been noticed that the oscillation frequency value is affected by an old solution. The other stock solutions are prepared in high amount and stored for several days: ferroin solution [0.88 mL, 10^−3^ M in water]; sulphuric acid [1.25 mL, 1 M in water]; malonic acid [1.67 mL, 1 M in water]; and potassium bromate [1.65 mL, 0.5 M in 1 M sulphuric acid]. Reagents are mixed in the reported order, the amounts correspond to a typical experiment and will yield an oscillation period around 20 s. Different starting periods can be obtained by changing the amount of potassium bromate. During each experiment the webcam data stream is analysed by the pcDuino in order to extract the oscillation period and make real-time decisions. The count of blue pixels is also saved in a.csv file on the board for post processing (Supplementary Fig. 10). Due to the limited computational power of the pcDuino for real-time analysis the frame rate is set on 3 fps, it is considered an acceptable speed since each BZ oscillation lasts for about 2–3 s. By using real-time additions of water and potassium bromate two BZ reactions have been successfully synchronised in real time using Twitter. In Fig. 5a it is possible to observe the two boards starting with different oscillation periods: around 20 s the leader (red line) and around 65 s the follower (blue line). As soon as the reaction starts to oscillate the leader board begins to extract its own oscillation period through the webcam and tweet it every 4 min. After 40 min the follower starts to monitor its own period in the same way and to check the leader’s period. By using the empirical functions (see Supplementary Method 3.3), the algorithm makes an estimation of the amount of starting material to add in order to synchronise its period with the leader’s one. Within few iterations the two periods are synchronised with an uncertainty of 2 s. The period of both platforms are recorded for the next hour and half showing that the reactions keep oscillating at the same frequency. For message encoding/decoding the string is first splitted into individual characters and each is converted into a number using an optimised alphabet, from 0 to 63. The number is converted into octal numerical system (base-8 numbers), the obtained octal is expressed using the degree of modulation of the reaction frequency. To represent an octal base there are eight thresholds for oscillation period: 21, 15, 9, 3, −3, −9, −15, and −21 s. The alphabet is designed to associate the most common letters (i.e.: a, e, t) with the smaller period gaps. We noticed that the oscillating reaction becomes less reliable after a series of large changes (±21 s difference). Therefore, having the small amounts being chosen more often leads to a higher reliability in the message encryption process. Two reactions are started with random amounts of Potassium bromate and synchronised after 10 min using the described procedure. Then the ‘Leader’ board starts adding the materials amount corresponding to the message, each addition is calculated in real-time in order to obtain the desired period difference and it depends on the current reaction period. 4 additions are performed and sent in sequence to the ‘Follower’ board every 7.5 min. The ‘Follower’ adds the material, reads the periods and decodes the message.

### Inorganic

The polyoxometallate chosen for this study was previously reported within our group and can be seen in Fig. 6b. Formula: [W_19_M_2_O_61_Cl(SeO_3_)_2_(H_2_O)_2_]^9−^. Reagents are stored in stock solutions: Na_2_WO_4_.2H_2_O (75 g), Na_2_SeO_3_ (6 g), DMA.HCl (30 g) in 450 mL; MnCl_2_.4H_2_O (9.36 g) in 450 mL; and 2.32 M HCl (57.1 mL conc made to 300 mL). Reaction series of varying W/Se:Mn ratios were performed collaboratively by two platforms communicating in real time over Twitter/shared server. Within each series 15 reactions of varying pH were used to determine the ideal conditions for producing crystals within a 2-h window. Later successful conditions were collaboratively repeated to assess the reproducibility of crystal formation. Peristaltic pumps supply volumes of stock solutions to a reaction vial (Supplementary Method 4). Following 10 min of stirring at room temperature each reaction solution was transferred to a recording vial suspended over a HD webcam, again using a peristaltic pump. Reactions are recorded and analysed in real time for crystal formation and results are stored both locally and on a shared network. Once complete both reaction and recording vials are extensively cleaned with an automated cycle for the process to begin again. Image analysis techniques were developed to allow for full automation of reaction monitoring for crystal recognition in real time via a HD webcam. A hand-annotate image data set of clear and crystallised reactions was prepared in order to allow a machine-learning algorithm to learn from a statistically significant sample set (i.e. 1295 image samples for both clear and crystallised reactions). The learning algorithm was formulated as a binary classification in order to recognise between clear and crystallised reaction states. The classification consisted of the visual Bag of Words (BoW) technique^21^. BoW is about encoding relevant topics into distinctive bags containing ‘visual words’. Each bag groups features that share colour similarities; hence a collection of bags represents the vocabulary knowledge that can be used for classification and recognition.

The successful recognition between reaction states is predicated on the representational richness of the visual description. For this, we computed a global image descriptor based on two-dimensional colour histograms between the hue, saturation and value (HSV) channels, encoding the colour statistics of a video frame into 75 bins. Each descriptor was later normalised to achieve illumination invariance and improve the recognition between reaction states. In all, 1295 image descriptors were obtained for each image sample in the data set. Then, the *K*-means clustering algorithm was applied to partition the visual vocabulary into 100 clear and 100 crystallised clusters. The latter allowed us to obtained a compressed visual description of the data set, which was then used to train a binary support vector machine (SVM). We tested the SVM classification model using 10-fold cross-validation where a mean classification accuracy of 95% of recognising the correct reaction state was obtained. A frame of the reaction solution is taken every 8 s and analysed for the presence of crystals using this model. Once a specific threshold of frames containing crystals has been met the programme determines the reaction a success and updates the network. If no crystals have been observed after 2 h the platform starts a cleaning cycle and continues with the remaining reactions. Further image processing of the early reaction solutions using colour analysis (hue) is deployed to identify precipitated reactions. Precipitation of this reaction occurs most often within the first few minutes of the reaction recording. After 5 min of reaction recording a frame is analysed using this method. A mask is applied to isolate the reaction solution, a colour conversion from RBG (red, blue, green) to HSV is applied and largest representative hue value is returned. A hue value above 160 in all cases seen has been precipitates. This method is highly reliable and saves a great deal of time running the platform long term. Simple python commands are used to perform and report on the reactions to a network.

### Gameplay

The stock solutions and chemistry used in this section were the same as the methods in the Organic section above. Details of decision making, strategy and real-time platform communication can be found in Supplementary Method 6.

### Code availability

Software to run the system is available at https://github.com/croningp/NetworkedChemistryPlatform.

## Electronic supplementary material

Supplementary Information

## Data Availability

The authors declare that data supporting the findings of this study are available within the paper, its Supplementary Information files and on request from the authors.
