# Supplementary material for: Networking chemical robots for reaction multitasking
Source: Nat Commun. 2018 Aug 24;9:3406. doi: 10.1038/s41467-018-05828-8 (PMC6109044; doi:10.1038/s41467-018-05828-8)
Supplement: Supplementary file 1 — Supplementary Information [file 41467_2018_5828_MOESM1_ESM.pdf]

Supporting information

Networking Chemical Robots for Reaction Multitasking

Dario Caramelli, Daniel Salley, Alon Henson, Gerardo Aragon Camarasa, Salah Sharabi, Graham Keenan, Stuart Marshall and Leroy Cronin*

WestCHEM, School of Chemistry, The University of Glasgow, Glasgow G12 8QQ (UK)

1. Robot design and concept: ... 2
 1.1 Peristaltic pumps ... 4
 1.2 pcDuino board ... 4
 1.3 Power supply unit ... 5
 1.4 Software ... 5
2. Part I – Organic ... 7
 2.1 Organic chemical space ... 7
 2.2 Colour detection ... 8
 2.3 Collaborative algorithm ... 9
3. Part II – Physical ... 11
 3.2 Plot the oscillations ... 11
 3.3 Predicting the chemical influence on oscillation period. ... 12
4. Part III – Inorganic ... 15
 4.1 Stage one- Collaboratively explore a chemical space ... 15
 4.2 Stage two- Repetition of Successful conditions ... 16
 4.3 Grid search of reaction conditions ... 17
5. Agent based simulation ... 20
6. Game ... 22
 6.1 General Overview ... 22
 6.2 Decision Making ... 22
 6.3 Communication Between Platforms ... 22
 6.4 Strategy ... 23

Supplementary methods

1. Robot design and concept:

The robot computational core is a pcDuino3 running Linux Ubuntu operating system that executes homebuilt code in python to control a number of pumps and a webcam. Access to internet is achieved via a wired ethernet or WiFi connection. For everyday use the board was connected also to a monitor, mouse and keyboard. Liquid handling is performed by a set of peristaltic pumps, the pumps are turned on for duration of the required addition time. The pumps are connected through tygon® tubing with the reagents and the reaction flask. Generally 5 pumps are dedicated to adding the reagents, one adds water for washing and the last one is used to empty the reaction flask. Data is aquired with a USB webcam able to record images and video from the reactions. The reactions are performed in a standard 14ml glass vial. It is magnetically stirred with a home built stirrer using a small fan. The robot has been designed to be as simple and affordable as possible. Therefore it can be assembled in just few hours.

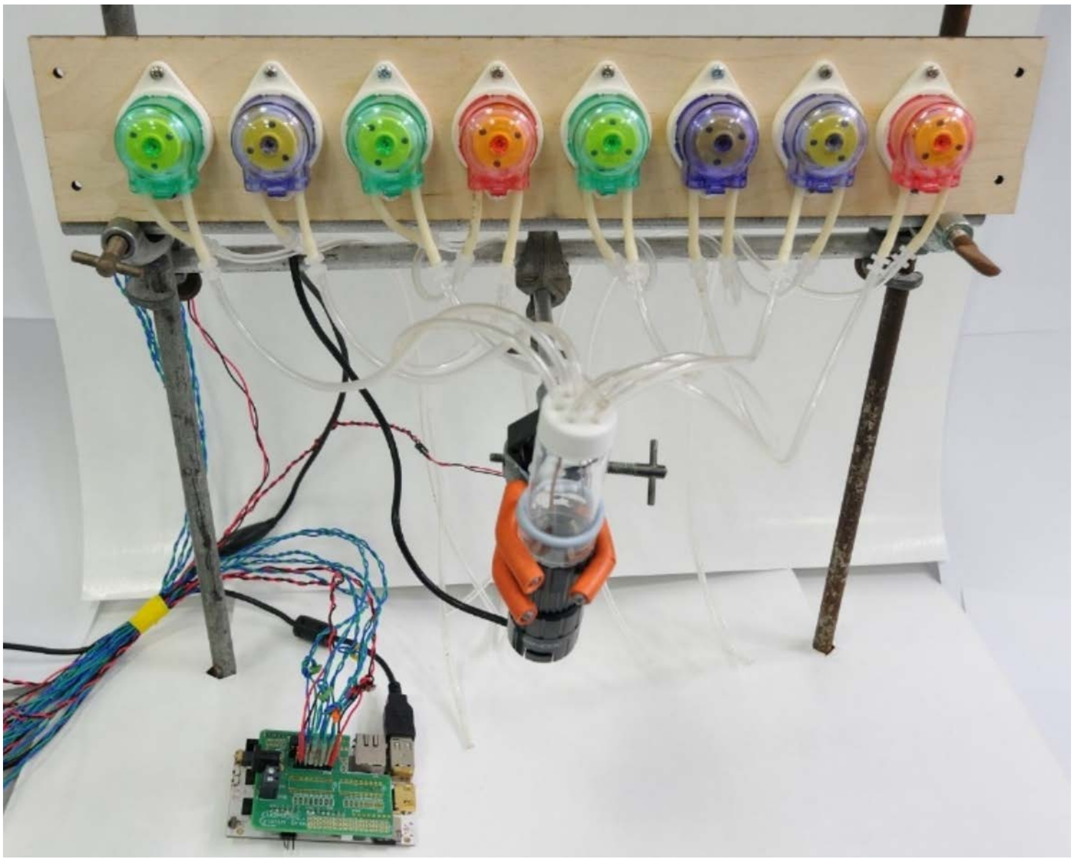

Supplementary Figure 1: Photograph of the Robot. A unit is made of a set of peristaltic pumps for liquid handling (top), a webcam for reaction analysis (center, under the vial), and a pcDuino board for electronic control (bottom left).

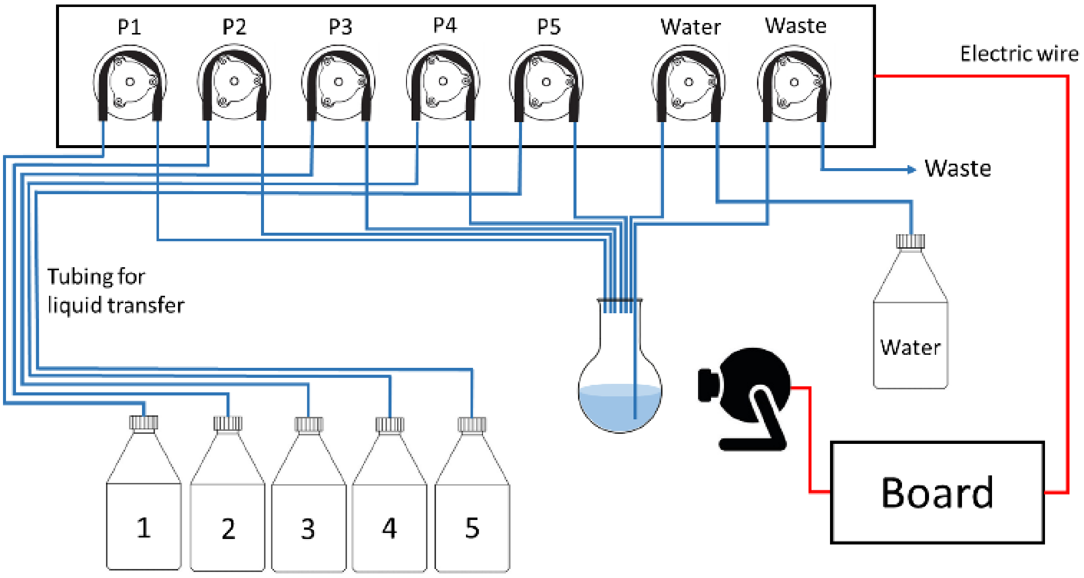

Supplementary Figure 2: Schematic representation of the platform. Electric connections are colored red and liquid transfer tubing connections are colored blue.

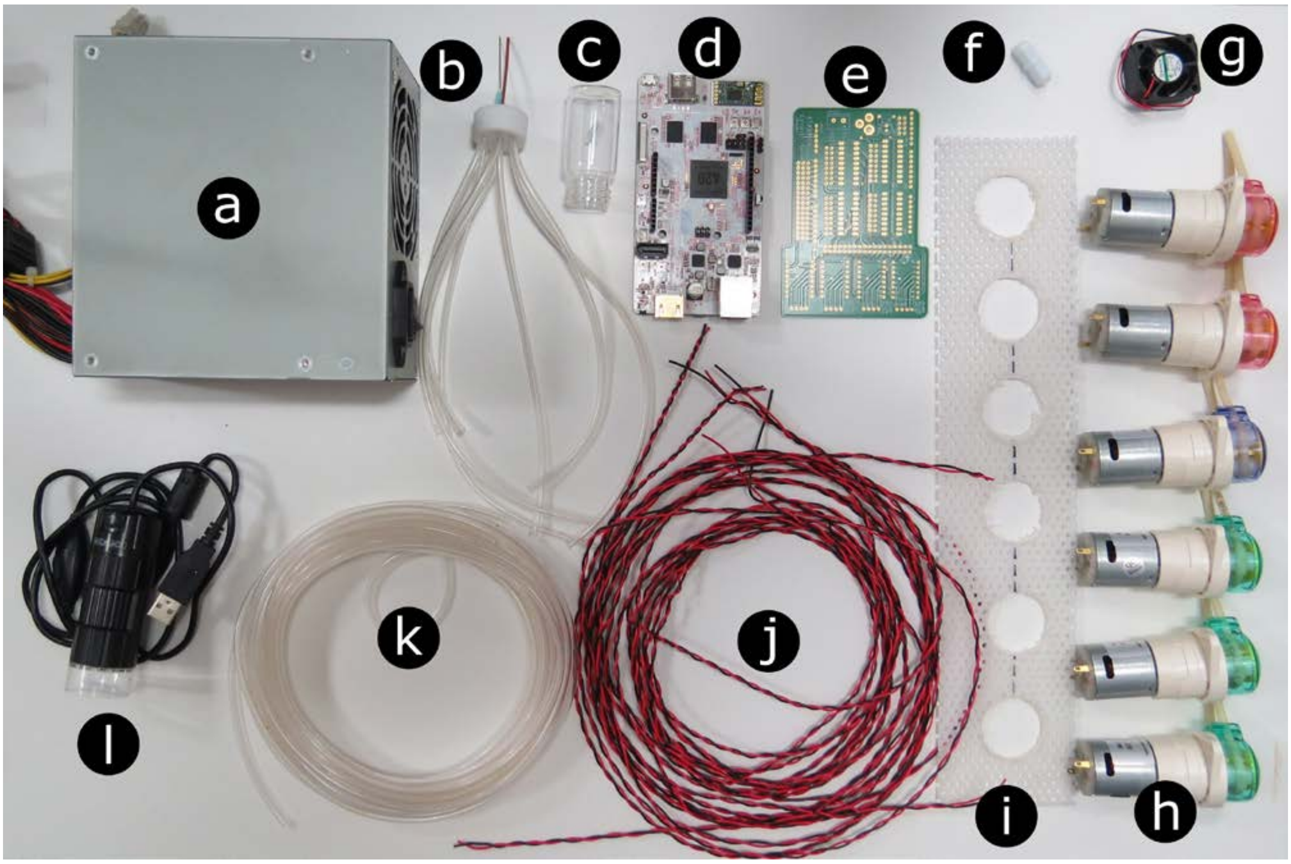

Supplementary Figure 3: Robot required parts. **a)** Power supply (12V,5V), **b)** vial cap with tubing, **c)** a glass vial, **d)** pcDuino board, **e)** costum designed pumps driver board, **f)** magnetic stirrer bar; **g)** small dc fan, **h)** peristaltic pumps, **i)** pumps holder panel, **j)** electrical wiring, **k)** Tygon® tubing, **l)** USB webcam

1.1 Peristaltic pumps

The control over the solutions was performed using a set of peristaltic pumps. The pump is driven by a 12V DC and it is connected to the driver board mounted on pcDuino. In this work, we used the model KFS-HB2B06M, where M is either R,B,G,P which refer to pump colour (Red,Blue,Green,Purple). The pumps are designed to have a flow rate of 4ml/min towards a single direction. Since a loss in precision over time was observed the pumps were recalibrated every week and after any manteinance operations.

1.2 pcDuino board

The robot runs on a pcDuino3, it is powered by a 5V (2A) power supply fed through a micro USB cable. This board features the following:

- CPU: AllWinner A20 SoC 1GHz ARM Cortex A7 Dual Core
- GPU: OpenGL ES2.0, Open VG 1.1 Mali 400 Dual core
- 1GB DRAM, Onboard Storage: 4GB Flash memory, microSD card slot (supports up to 32GB)
- Arduino style Peripheral headers
- HDMI Video output
- SATA socket, IR receiver, LVDS LCD interface and MIPI camera interface
- Audio out: 3.5 mm Analog Audio and I2S Stereo Digital Audio
- USB interface
- RJ45 Ethernet Connection 10M/100Mbps and Wi-Fi module

API interfaces such as UART, 6xADC, 2xPWM, 14xGPIO, 1xI^{2}C, 1xSPI

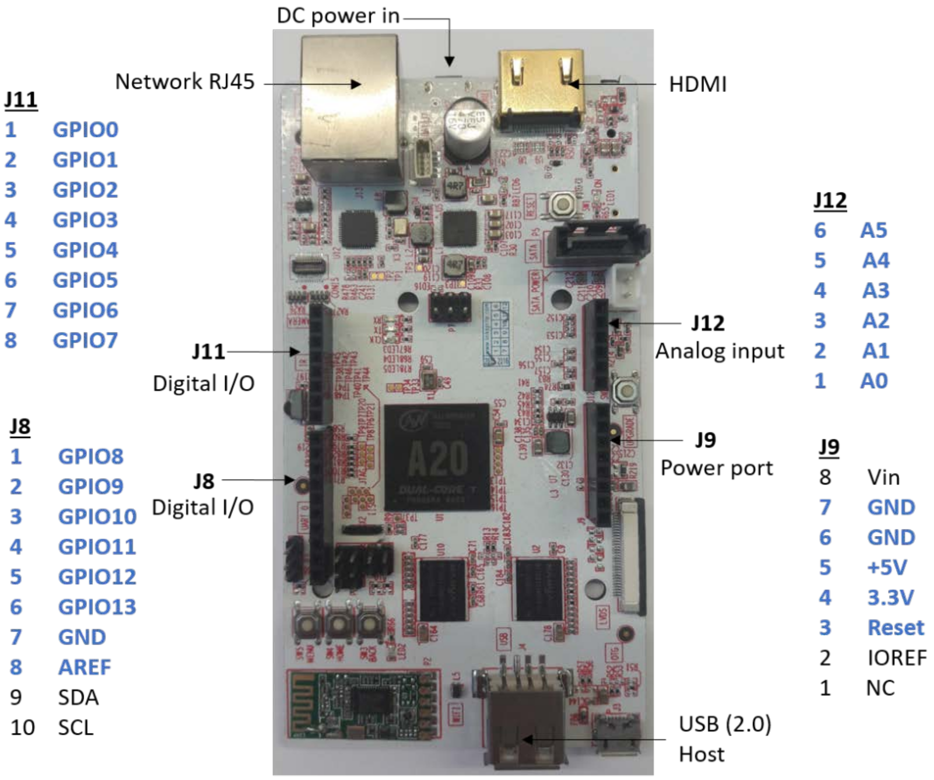

Supplementary Figure 4: PcDuino3 board.

1.3 Power supply unit

The robot is powered by a 5V (2A) DC power source. However, the Peristaltic pumps are driven by a 12V (1A) DC source. In this work, a 500W ATX power supply unit was used.

1.4 Software

The pcDuino3 runs with the Ubuntu operating system. The platform is controlled by a dedicated program written in python. Due to specific experiments each project part has been completed by using a dedicated program. However, the low-level software is the same and is composed by three main parts with respective external libraries:

<u>Pump control:</u> This is based on gpio, a common library to control the pins of the pcDuino, and therefore operate the pumps. Since there is no feedback from the pumps a code converts the amount of solution required into a time interval used to run the pump. This time interval is derived from a calibration process where the flow rate of each pump is tested, verified and saved as a variable.

<u>Webcam control:</u> This is based on the OpenCV library. The webcam is accessed by the computer and provides images and videos of the reaction. Further image/video analysis will be discussed in the respective project sections.

Network management: This uses the Twython library and controls the networked part of the platforms. It allows the platform to update its state by sending a tweet on its account and scan other accounts for synchronization and collaboration.

Coordinator: The software core of each project section is a “coordinator” program. It manages all the experiment components: physical reactions, image analysis, network synchronization and search algorithm.

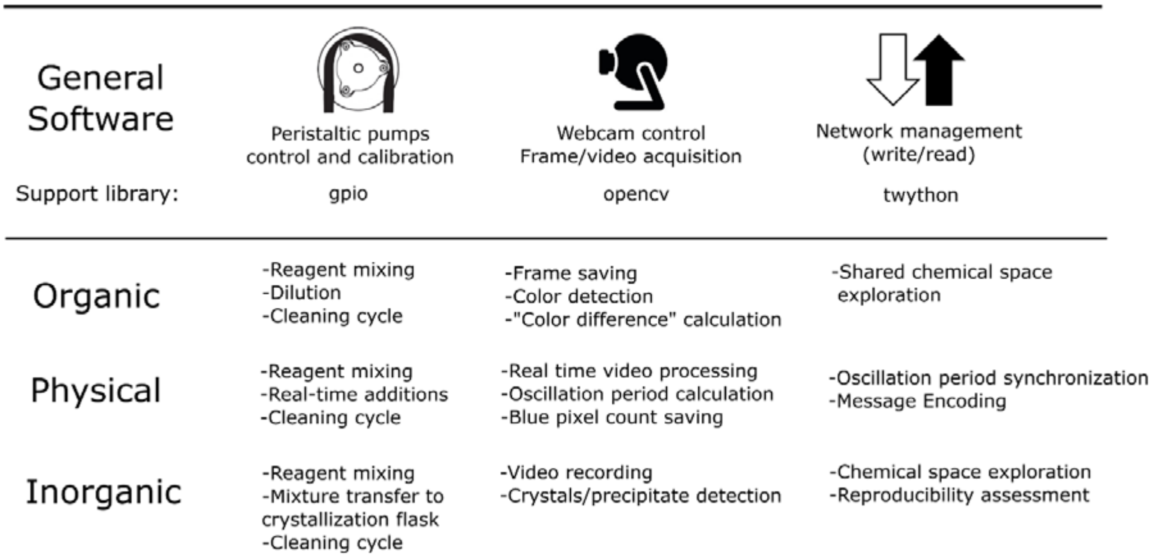

Supplementary Figure 5: Description of the general operational software. At the top the common and lower layer code is reported. Based on that we developed specific programs for each project. They will be discussed in the relative sections.

2. Organic

2.1 Organic chemical space

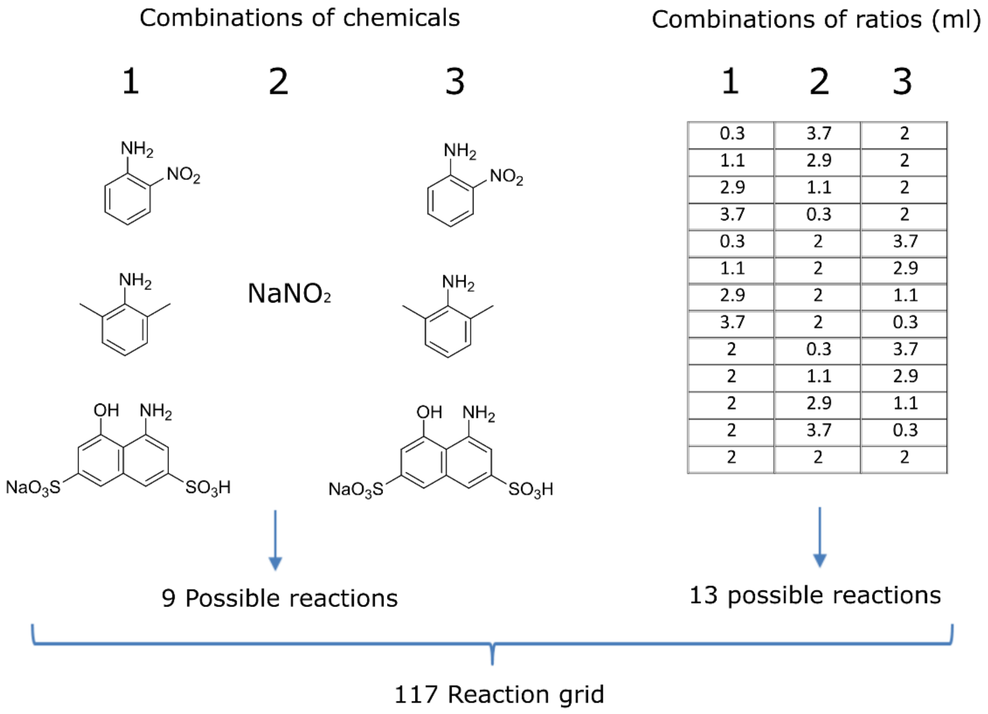

1	2	3
0.3	3.7	2
1.1	2.9	2
2.9	1.1	2
3.7	0.3	2
0.3	2	3.7
1.1	2	2.9
2.9	2	1.1
3.7	2	0.3
2	0.3	3.7
2	1.1	2.9
2	2.9	1.1
2	3.7	0.3
2	2	2

Supplementary Figure 6: **Organic reaction grid.** Structure of the expanded chemical space as a combination of reagents and ratios.

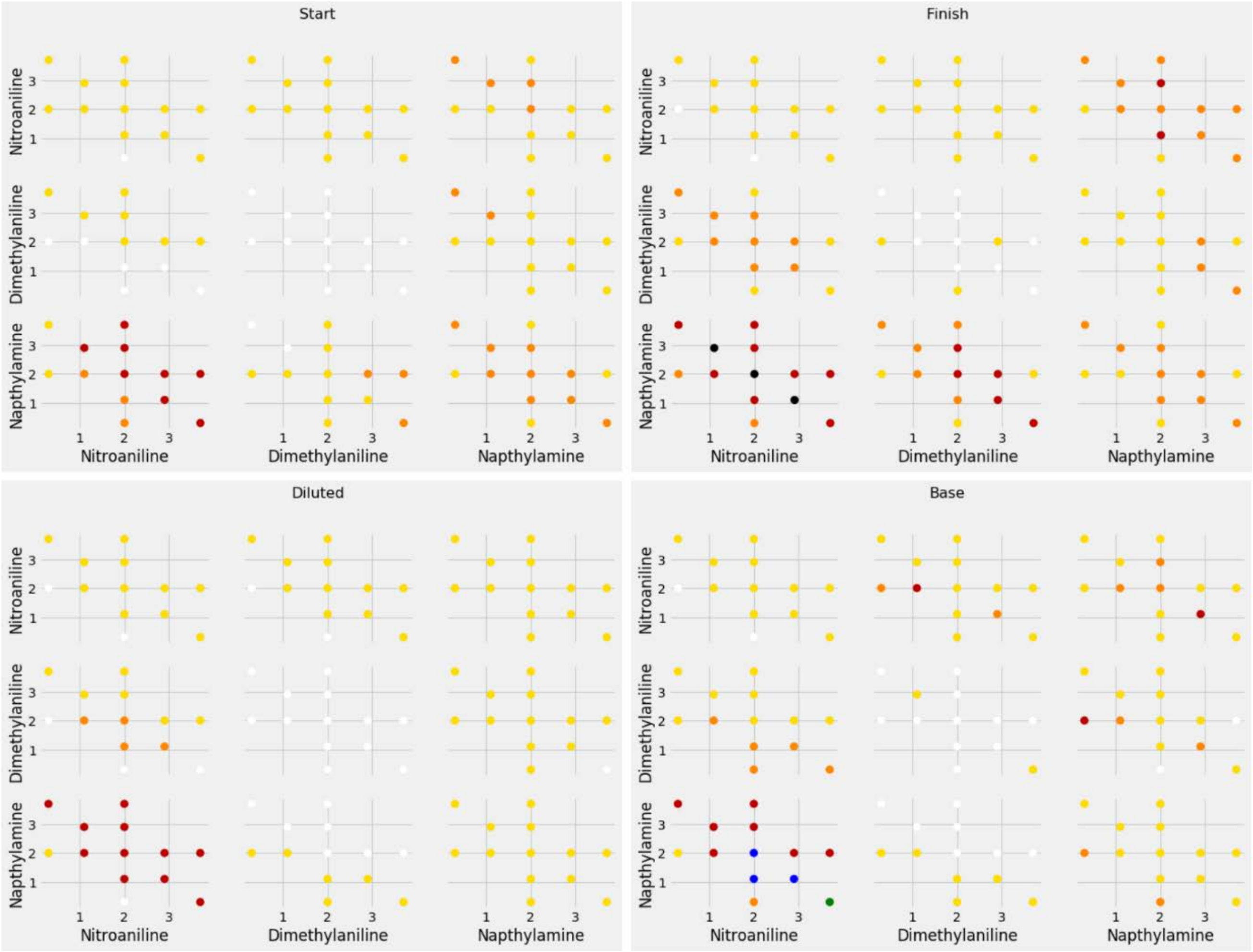

Supplementary Figure 7: **Four colour plots showing the full 117 reaction grid**. Each plot shows a specific reaction moment. X axis corresponds to the aniline derivate used as first, Y axis to the one used as second. Subplots show 13 combination of ratios, the colour of the point corresponds to the solution colour extracted with the webcam.

2.2 Colour detection

For the colour determination, the image frames recorded are converted into hsv colour domain. To allow the calibration of each colour, they have to be associated to a specific hsv range value. In each experiment a region of interest is analyzed and the pixel values are compared with the color ranges. The colour with the highest pixel count is considered the solution colour (red, orange, yellow, blue, colorless, black). Individual colour counts are saved and stored in a csv file for post processing.

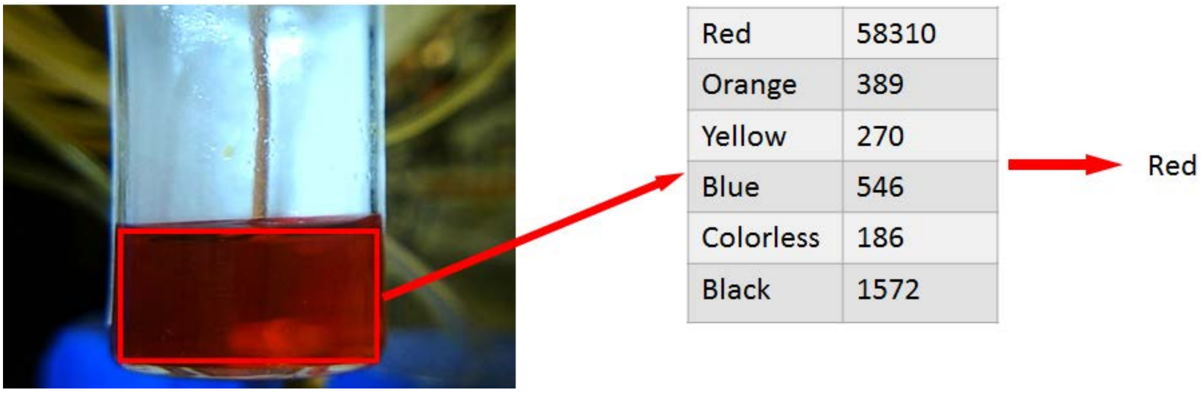

Supplementary Figure 8: Automatic colour detection. Example of frame recorded with the webcam, from a region of interest the pixel count is extracted. The solution colour corresponds to the highest value.

2.3 Collaborative algorithm

Two identical and physically separated platforms have been used to explore the 117 reaction grid. They run the same algorithm and the aim was to find a blue reaction using a random search, sharing the results in real time using Twitter to reduce total time. The algorithm starts by selecting a random reaction and sending a Tweet with the reaction parameters. The system then performs the selected reaction and saves 4 frames. These are analyzed on board, the database is updated and an "end" Tweet with the results is sent. If a blue reaction is not present in the database the board will restart with a new random reaction, otherwise it will send a "stop" Tweet and stop. A separated thread in the background checks every 5 minutes the other board's Tweets and update the database with those reactions result. In this way both boards will avoid performing the same reaction twice.

This script has been used to look for a blue reaction out of 117 total combinations. After 14 sequences blue has been found on average after 15.1 reactions. The theoretical number of reactions necessary for two platforms sharing results looking for 3 blue reactions out of 117 is 19.5.

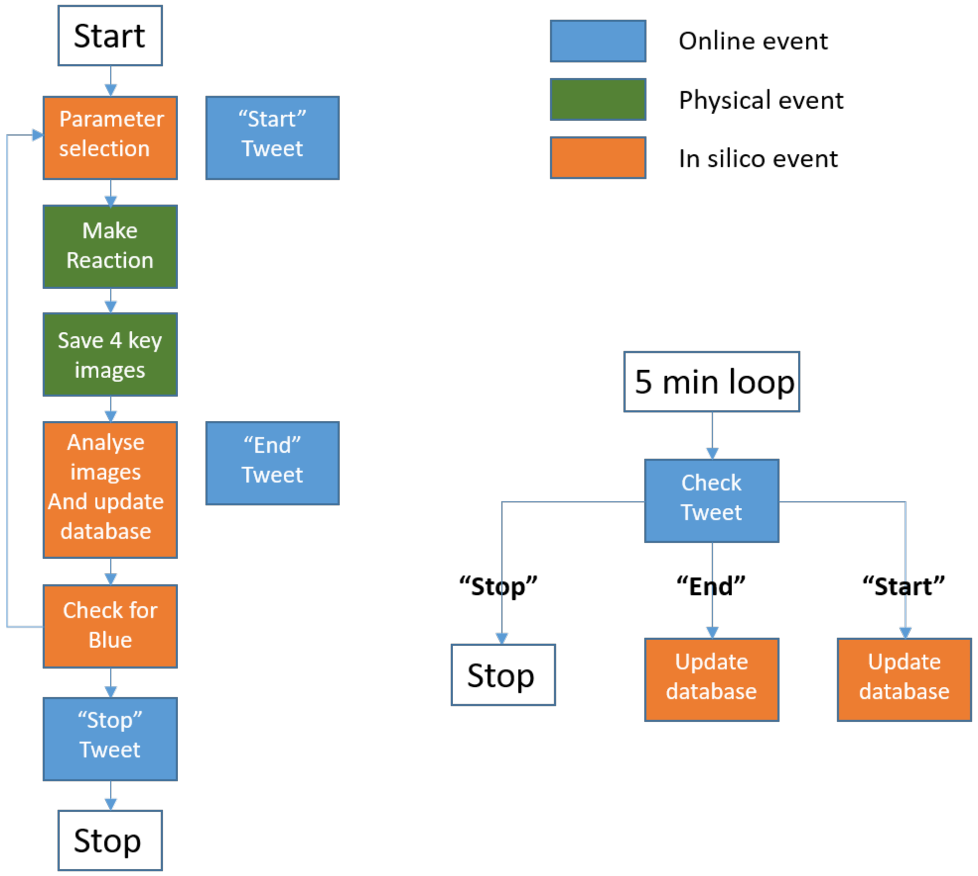

Supplementary Figure 9: **Collaborative algorithm run by two identical platforms**. It has a main thread (left) that manages the reaction making, data saving/analyzing and result sharing. The second thread (right) checks every 5 minutes the other board results and updates the database.

3. Physical

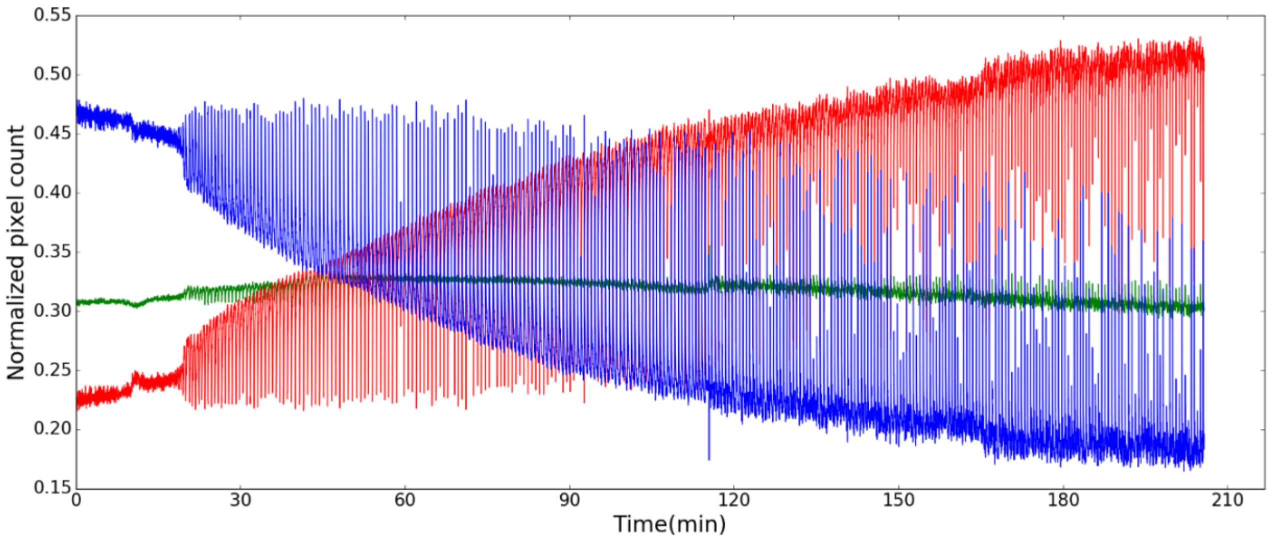

Supplementary Figure 10: **Recording of 3 hours BZ reaction.** Normalised red green and blue pixel counts are plottet vs time. Green pixels are approximately constant for the whole reaction. Oscillations start around 20 minutes. Until the end of the reaction (200 minutes, 3.3 hours) it oscillates around 540 times.

3.2 Plot the oscillations

In order to observe the oscillation period behaviour over time a script for data processing was created and the output demonstrated in Supplementary Figure 11-bottom.

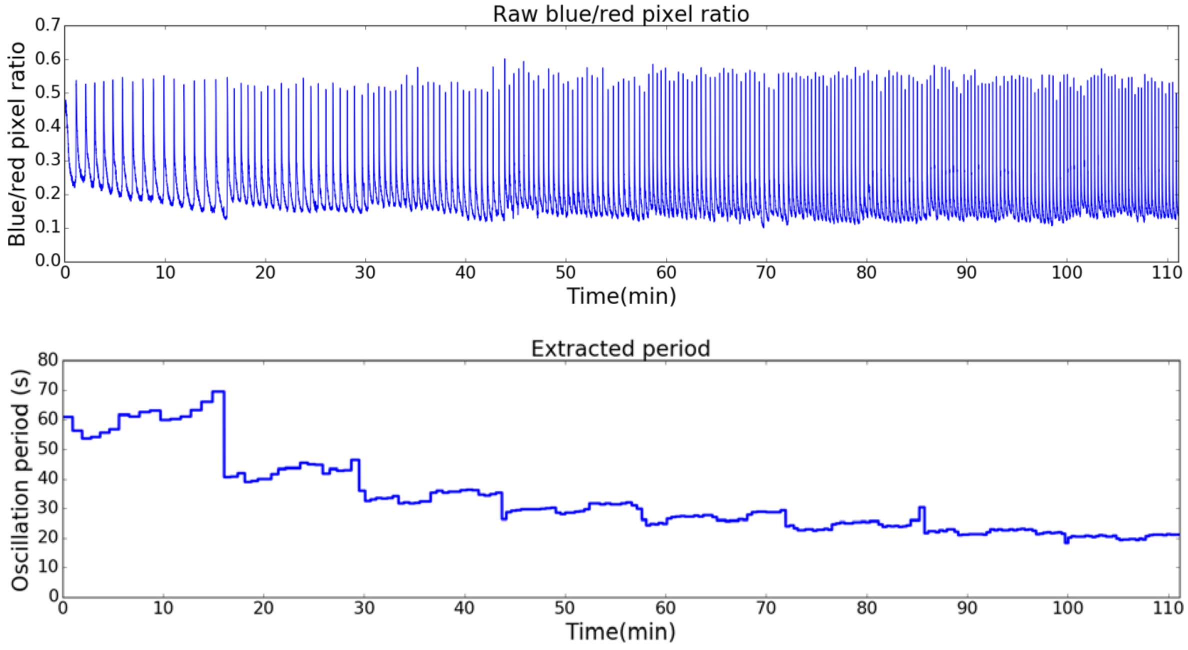

Supplementary Figure 11: Example of period extraction. Top: Raw data of blue\red pixel ratio on an oscillating BZ reaction recorded with the webcam. Bottom: same data after the period extraction processing.

3.3 Predicting the chemical influence on oscillation period.

In order to predict the behaviour of the oscillation period when small amounts of water and potassium bromate are added we monitored several reactions while constant and regular additions were made. By processing the results, it has been possible to obtain two functions that correlate the amount of material added with the oscillation period change, within a reasonable time window and error.

Water additions – slower period

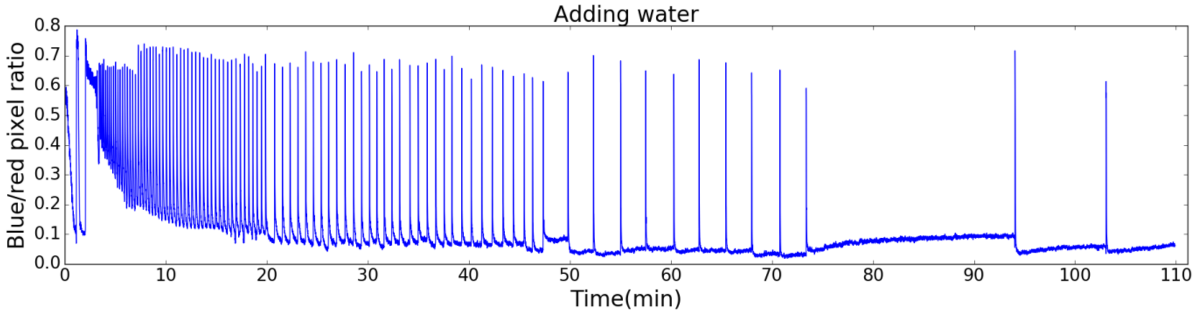

Supplementary Figure 12: **Example of water additions to an oscillating BZ reaction causing slower periods**. After 20 minutes from reagents mixing 1 ml of water is added. This is repeated at 48 and 73 minutes.

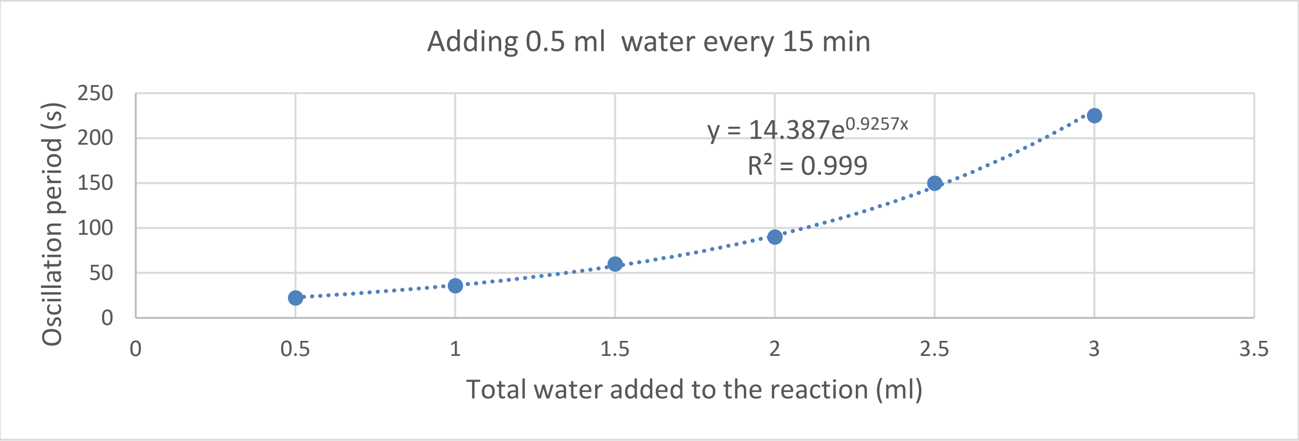

Supplementary Figure 13: Correlation of oscillation period and water addition. 0.5ml of water are added every 15 minutes to an oscillating BZ reaction. The graph shows the extracted oscillation period and its dependence to the amount of water added.

Shape of the curve is: $period = k * e^{amount}$ (equation 1)

Reversed form: $amount = \ln\left(\frac{goal\ period}{k}\right)$ (equation 2)

It will give an estimate of water *amount* to add in order to reach a specific *period*. Since it is referred to the reaction start, for real-time additions we need to consider also the current period:

$$amount = \ln\left(\frac{goal\ period}{k}\right) - \ln\left(\frac{current\ period}{k}\right) \text{ (equation 3)}$$

It easy to see that the empirical constant k is irrelevant, the function used to predict water additions is:

$$amount = \ln(goal\ period) - \ln(current\ period) \text{ (equation 4)}$$

When bromate is added there is a faster period, see Figure 18 and 19.

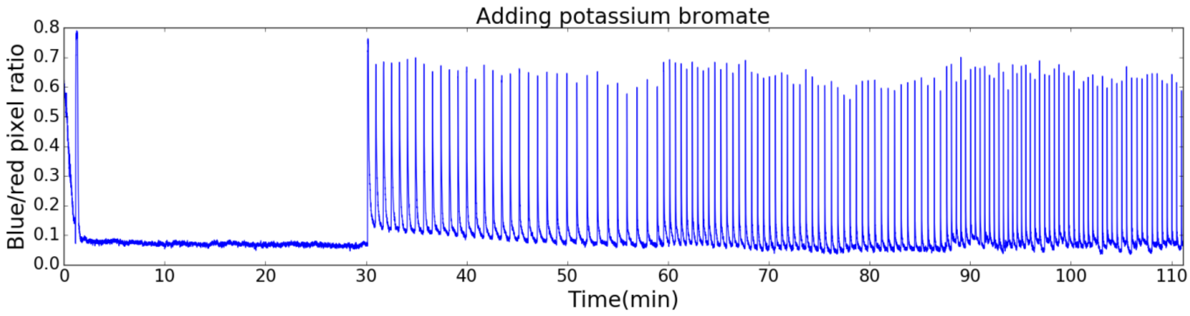

Supplementary Figure 14: **Example of potassium bromate additions to an oscillating BZ reaction resulting in faster periods**. After 30 minutes from reagents mixing 1 ml of potassium bromate is added. This is repeated at 60 and 90 minutes.

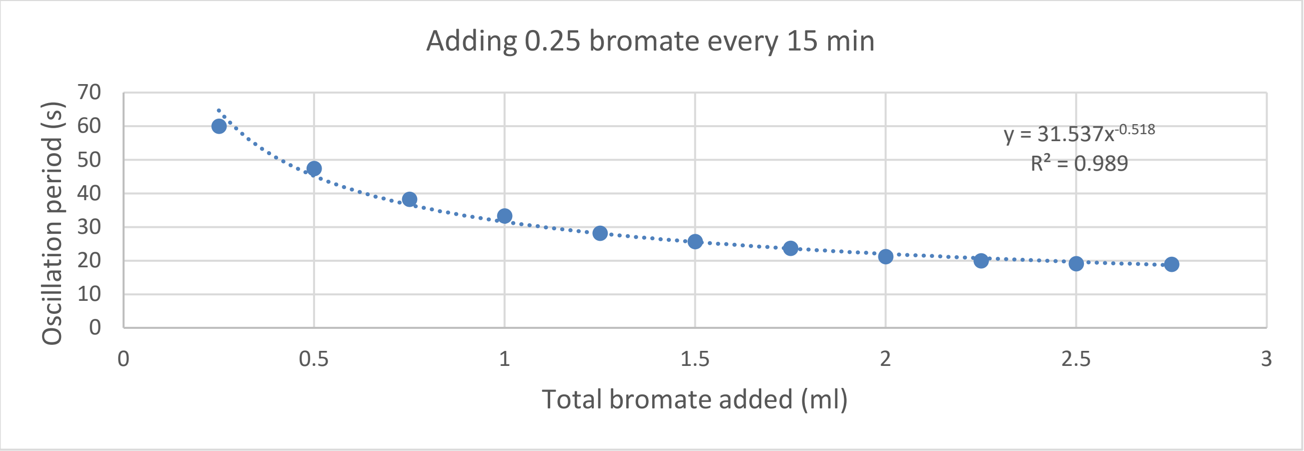

Supplementary Figure 15: Correlation between oscillation period and bromate addition. 0.25ml of potassium bromate are added every 15 minutes to an oscillating BZ reaction. The graph shows the extracted oscillation period and its dependence to the amount of potassium bromate added.

By using the data obtained in multiple addition tests we obtained the first empirical function

$$amount = \frac{num\ value}{current\ period^2 - goal\ period^2} \text{ (equation 5)}$$

Since the *numerical value* is not constant but depends on the period difference, a series of real additions at different periods have been used in order to obtain this correlation (**Supplementary Figure 16**).

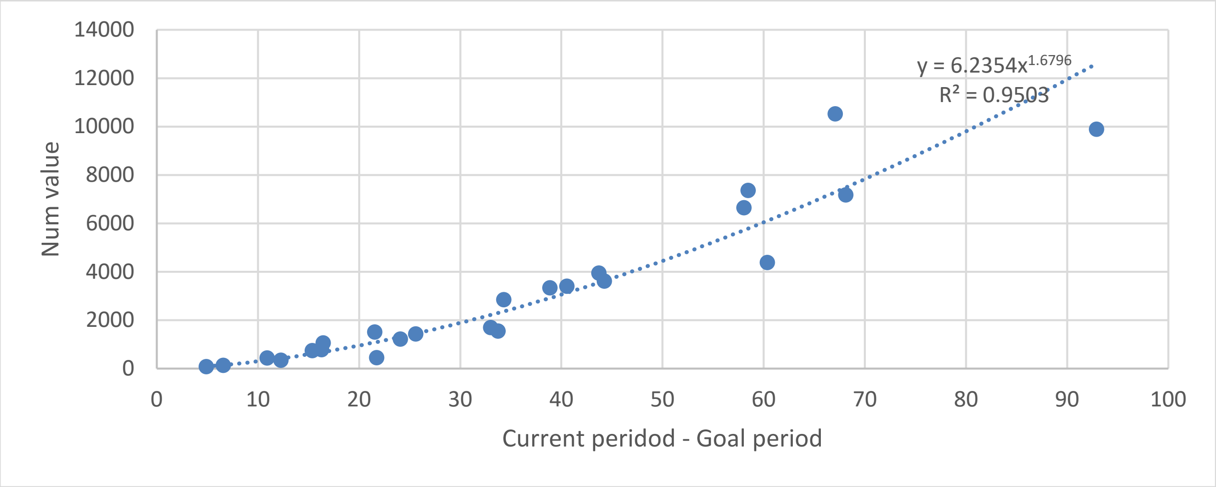

Supplementary Figure 16: correlation between numerical value of empirical formula and period difference. A series of real additions have been performed recording amount of material added and observed start/end periods. The *numerical values* calculated from this data and equation 5 are plotted vs the period difference and a new dependence is found.

By replacing the *numerical value* with the dependence to the period difference we obtained the final empirical function for bromate additions:

$$amount = \frac{6.2356(current\ period - goal\ period)^{1.67}}{current\ period^2 - goal\ period^2} \text{ (equation 6)}$$

4. Inorganic

4.1 Stage one- Collaboratively explore a chemical space

At each stage of synthesis and analysis both platforms update shared network files for the other to read and proceed accordingly. When one platform selects a reaction volume at random to explore, the other will acknowledge this and remove it from its own series before continuing with its own choice. Conditions that have produced crystals are stored by both platforms for repetition later. The flow diagram (**supplementary Figure 17**) describes the collaborative process between two platforms, the leader and follower:

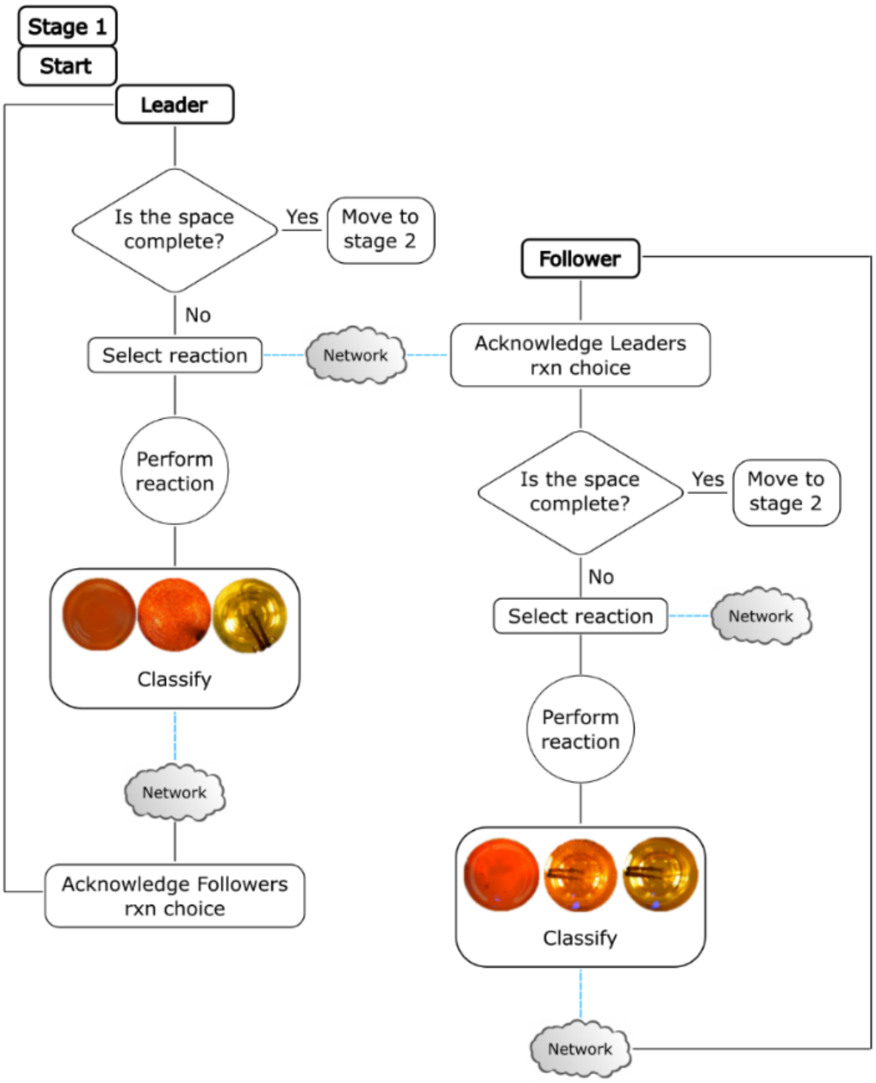

Supplementary Figure 17: Stage 1 of the algorithm for collaborative chemical space exploration. Different parameters are tested by two systems sharing the workload.

4.2 Stage two- Repetition of Successful conditions

The successful reactions conditions from the collaborative stage are compiled and repeated in order to establish the reporducibility of the chemistry/crystallization. One set of conditions is choosen and both platforms perform repeat reactions. Once enough data has been collected to establish an average percentage of reproducibility of obtaining crystals is complete and the next set of reaction conditions are begun, see **Supplementary Figures 18 and 19**.

Seen below is the outline of stage 2, Assessment of the reproducibilty of crystal producing reaction conditions.

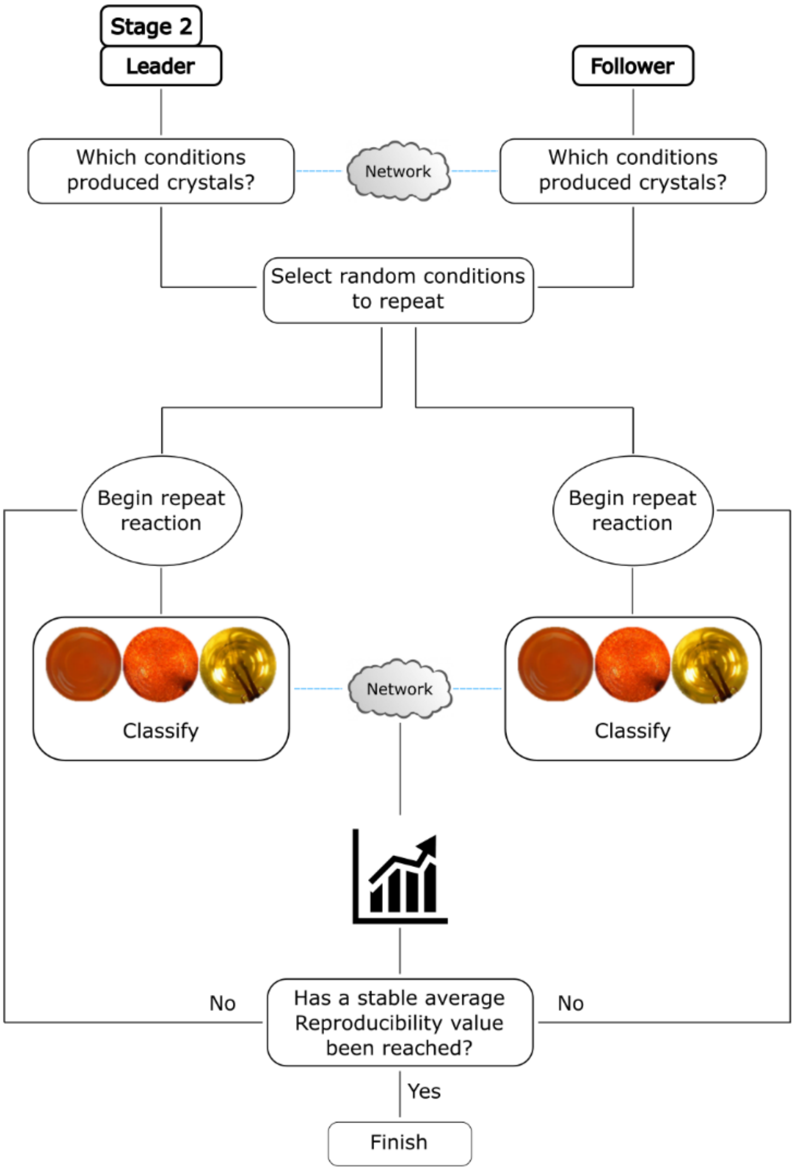

Supplementary Figure 18: Stage 2, Repetition algorithm for crystallizing condition. Repetition of crystal yielding reactions conditions to assess their reproducibility.

Below is included a timeline diagram of a single example of one such reaction cycle using a 1:6 W:Mn ratio. Constant communication between platforms + image recognition of precipitated reactions allowed for completion time of 20 hours 57 minutes.

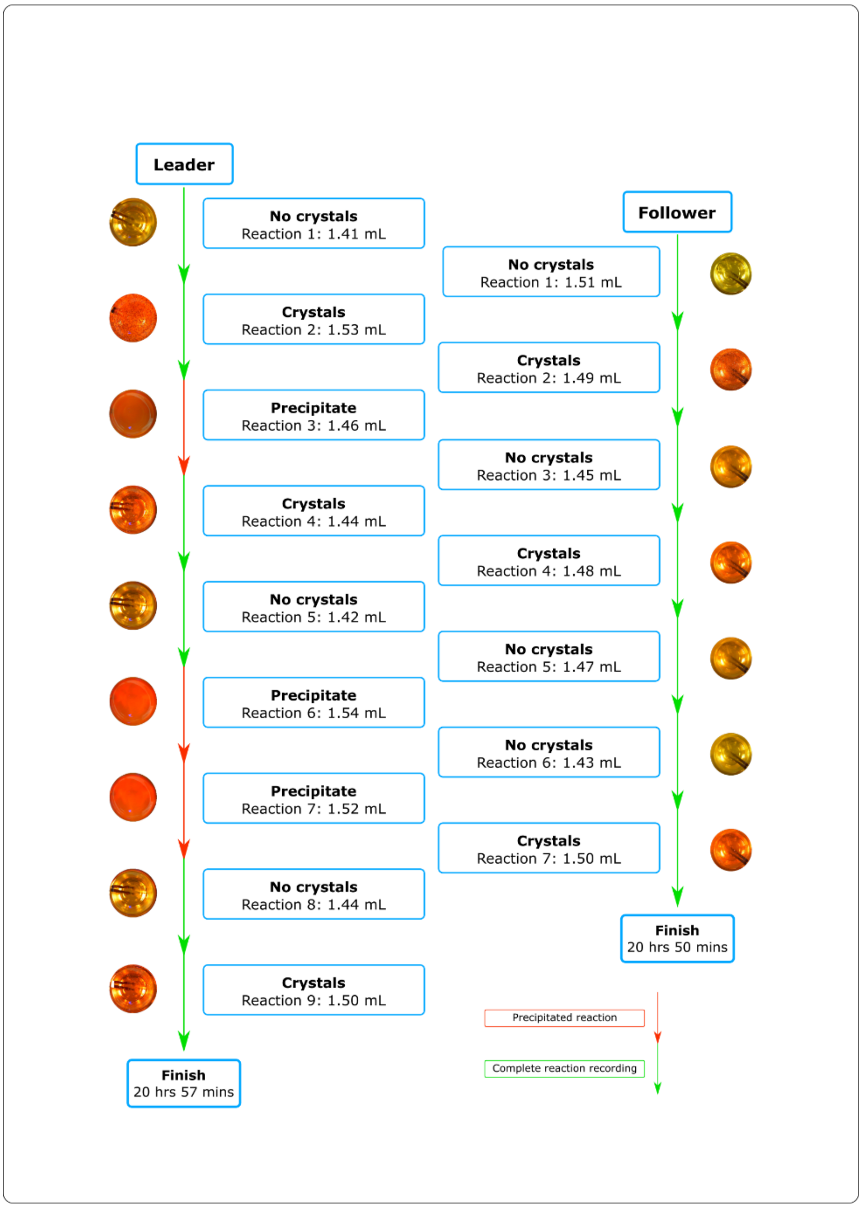

Supplementary Figure 19: Timeline of workload sharing between two platforms. Example of a 15 reaction series shared collaboratively between two platforms.

4.3 Grid search of reaction conditions

8 reaction series each with varying Mn:W ratio were performed collaboratively by the two platforms by the methods detailed above **(Supplementary table 1).** Each reaction series varied in acid volume from 1.4-1.54mL HCl (approximately between pH 3-6.5) and each reaction was monitored by web cam for crystal formation within 2 hours of reaction completion. The full grid was repeated 3 times

to more thoroughly explore the space. The results of each grid can be seen in below as 2D colour maps, see **Supplementary Figure 20**.

Ratio Mn:W	Acid Volume mL														
1:4	1.4	1.41	1.42	1.43	1.44	1.45	1.46	1.47	1.48	1.49	1.5	1.51	1.52	1.53	1.54
1:6	1.4	1.41	1.42	1.43	1.44	1.45	1.46	1.47	1.48	1.49	1.5	1.51	1.52	1.53	1.54
1:8	1.4	1.41	1.42	1.43	1.44	1.45	1.46	1.47	1.48	1.49	1.5	1.51	1.52	1.53	1.54
1:10	1.4	1.41	1.42	1.43	1.44	1.45	1.46	1.47	1.48	1.49	1.5	1.51	1.52	1.53	1.54
1:12	1.4	1.41	1.42	1.43	1.44	1.45	1.46	1.47	1.48	1.49	1.5	1.51	1.52	1.53	1.54
1:14	1.4	1.41	1.42	1.43	1.44	1.45	1.46	1.47	1.48	1.49	1.5	1.51	1.52	1.53	1.54
1:16	1.4	1.41	1.42	1.43	1.44	1.45	1.46	1.47	1.48	1.49	1.5	1.51	1.52	1.53	1.54
1:18	1.4	1.41	1.42	1.43	1.44	1.45	1.46	1.47	1.48	1.49	1.5	1.51	1.52	1.53	1.54

Supplementary table 1: **Inorganic reaction grid dispensing volumes.** 120 Reaction grid of W:Mn to HCl

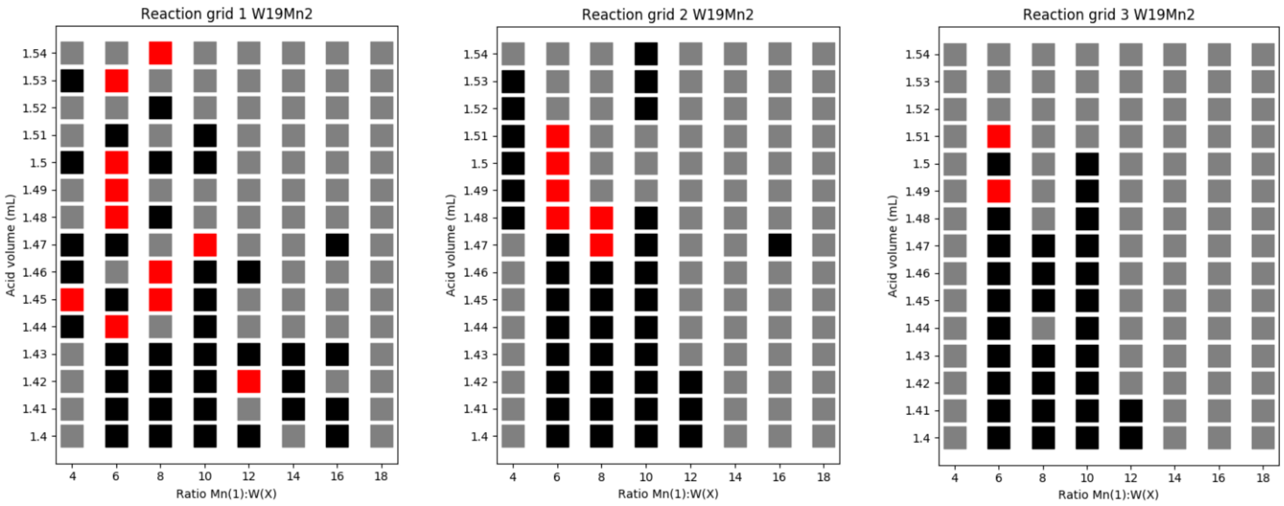

Supplementary Figure 20: Results of 3 repetitions of the full inorganic grid. Red squares correspond to the production of crystals, grey to precipitate and black to a clear solution.

Each of the conditions marked in red produced crystals at least once during these automated runs, all were repeated to assess the likelihood of growing crystals again. After 15 repeat reactions if no crystals had been produced the reaction was abandoned and the next set of conditions were started. A significant number of these crystalizing conditions never again produced crystals. Others varied from 10-50% in frequency of crystal formation across up to 48 reactions (**Supplementary table 2**). Shown below is a 3D representation of the likelihood of crystal formation for all conditions of the chemical space and the top 6 conditions for crystal formation without 2 hours, see **Supplementary Figure 21**.

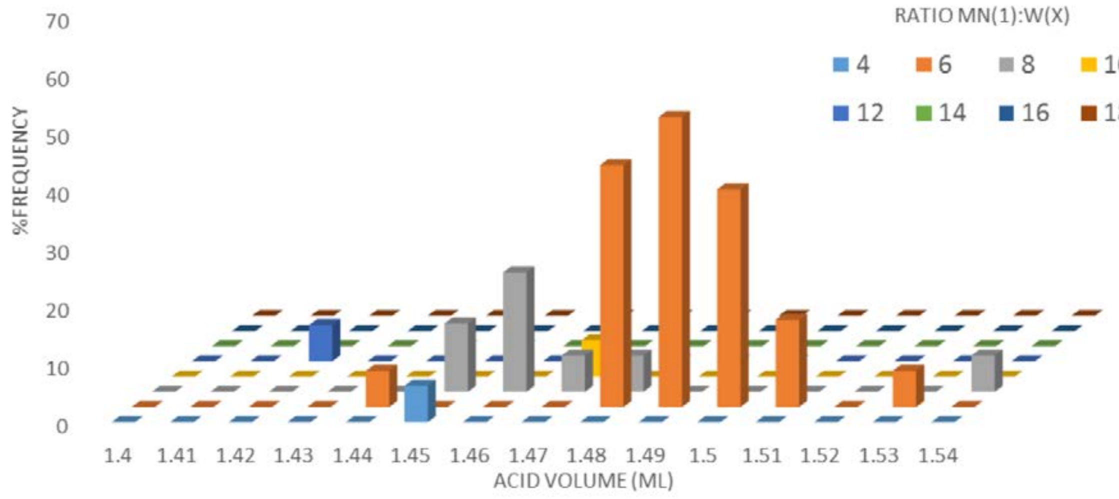

Supplementary Figure 21: **Bar chart of crystallization reproducibility.** Bar chart representation of the conditions found to produce crystals within a 2 hour window.

Ratio Mn:W	Total volume mL	Acid volume mL	Reproducibility %
**1:6**	10.38	1.49	50
**1:6**	10.37	1.48	41.7
**1:6**	10.39	1.5	37.5
**1:8**	9.35	1.46	20.6
**1:6**	10.4	1.51	15
**1:8**	9.34	1.45	11.8

Supplementary Table 2: **Hierarchy of crystallizing conditions**. Reaction conditions that produced the best reproducibility of successful crystal formation within 2 hours in descending order.

A comparison of the Leader and Follower platforms is included below in **Supplementary Figure 22**. The result distribution (no crystals, crystals and precipitate) of 150 repeat reactions were used to compare the performance of one platform to the other. A maximum 6% difference exists between crystal/precipitate formation reactions and a 1% difference in the number of reactions that produced no crystals shows a remarkable similarity between platforms considering the highly sensitive nature of the process under investigation and the cost of the platform.

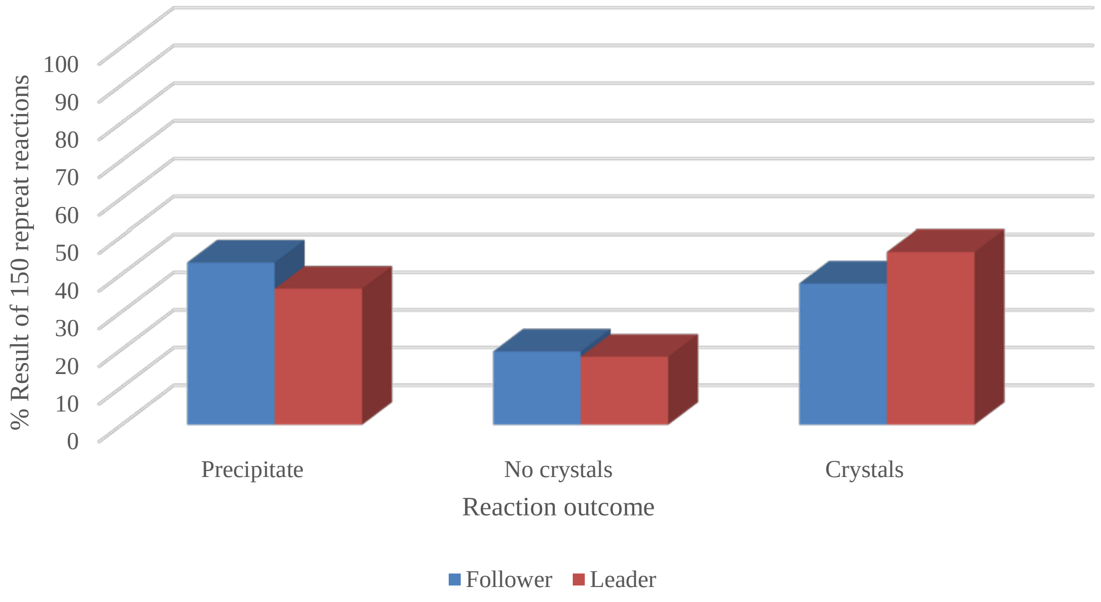

Supplementary Figure 22: Side by side platform comparison. A comparison of the Leader and Follower platforms reaction outcomes over 150 repeat reactions

5. Agent based simulation

To demonstrate the importance of information sharing between chemical robots we first developed computer simulations of the relevant strategies, see **manuscript Figure 1**. Each simulated chemical robot performs experiments randomly until the target experiment is conducted. The simulations are performed over three different approaches. The random strategy is the most basic, where the robots have no memory of the reactions they have conducted previously. As an example, if we take a space the size of 10 locations, the robot might randomly decide to look at location number 7 first, if the goal isn't there than it will choose another location to search. When making the decision this second time the robot does not remember that it had tried location 7 already without success and so is as likely to pick that location again as any other. The second strategy is called the individual strategy. In this case the robots remember their past. Thus for the example case above after trying location 7 the robot would not check it again. However, using this strategy one robot still acts independently as there is no information sharing between the robots. The final strategy is collaborative. Each of the robots knows its own and all the actions of all other robots. It is this pooling of information that makes the collaborative strategy the most favourable. **Supplementary Figure 23** shows the search efficiency as the average total number of searches that are performed before the goal was reached. For all cases the average number of experiments that need to be performed is the highest for the random strategy, better for the individual strategy and lowest for the collaborative. In the case of a single robot the collaborative strategy reduces to the individual. From that point as the number of robots increases the advantage of the collaborative strategy becomes more pronounced as the individual strategy becomes

less advantageous. The individual strategy is useful with a small number of robots but has diminishing returns with increasing numbers of agents. An increase of one robot from one to two yields a 50% improvement while an increase from two robots to three yields a lower improvement of 33.3% and so on. The reason of the performance constantly rising in **Supplementary Figure 23-right** is in the parallel search: when performing the searches in parallel with multiple robots although only one robot (most likely) reaches the goal the rest of the robots still perform their actions and these excess actions are therefore wasted. The amount of excess increases with the number of robots in use. It is important to note however that this is only an excess of actions so that the waste is one of resources. The time it takes to find the goal is always better with more robots and is unaffected by this latter issue. The simulations show that the collaboration strategy is by far the most efficient and that as the number of available robots increases the benefit of using collaboration increases as well. **Supplementary Figure 23-right** shows that for all strategies, the total number of searches that had to be conducted decreases. With the y axis logarithmic, the constant slopes show that the improvement in searching is exponential. The individual strategy will always be better than the random one, no matter the number of agents, yet by a narrowing margin. As expected all strategies improve with an increase in the number of robots yet the collaborative strategy is superior to both the random and individual strategy under any conditions.

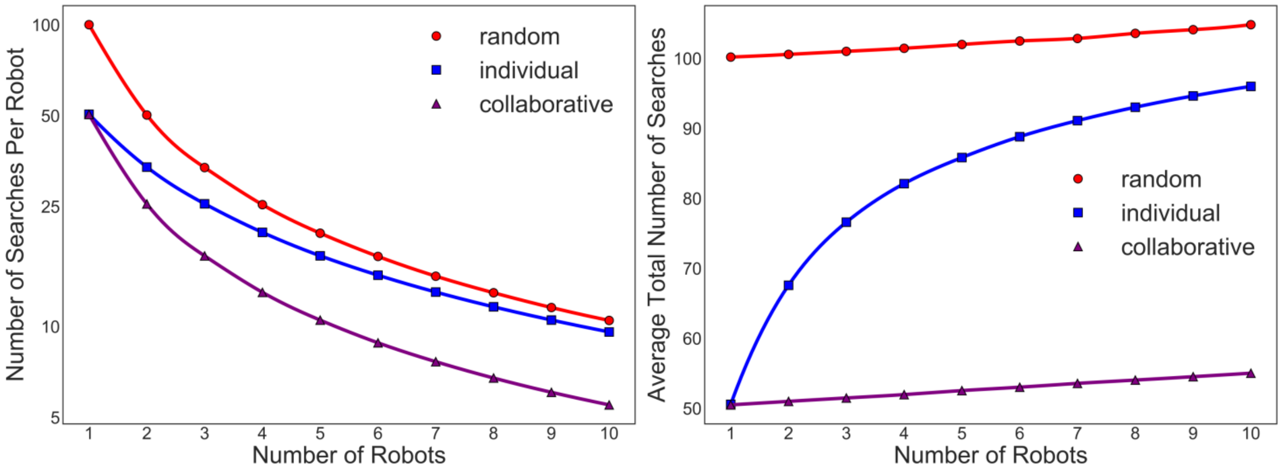

Supplementary Figure 23: Agent based simulations. On the left the number of searches for the three different strategies as a function of the number of agents conducting the search. The y axis is logarithmic. On the right the average number of searches that need to be perform in total for three different strategies with increasing numbers of agents.

6. Game

6.1 General Overview

Two automated platforms were tasked with playing a game of Hex. New/rare reaction results allowed the player to use the optimum movement (determined algorithmically described later) with uncommon/common results allowing only for sub-optimal/random movements. Losing games trigger a change in strategy for the losing platform, in this case an expansion of the reaction grid the player was allowed to explore. The idea being to show that a game outcome could drive a player to either change or maintain its current strategy in the hope of making more chemical discoveries in future. The chemistry chosen for this project was the same seen in the Organic section (**Methods part I Organic**) and results were gathered and analyzed via web-cam.

6.2 Decision Making

The goal for players in a Hex game is to connect one side of the board with the opposite side using a continuous line of that player's color. The game cannot end in a draw. From a randomly assigned first board position or the current state of the board the optimal movement was calculated using Monte-Carlo simulations with the goal of completing the game. Once an optimal movement has been calculated, the results of the chemistry determine if the player may use it. Color rarity vs move selection allowance is determined by the following:

- Unique/Rare colors observed up to 4 times — Optimal movement
- Uncommon Colors observed between 5 and 7 times — Sub-optimal movement
- Common Colors observed more than 7 times — Random movement

Sub-optimal movements were defined as a position beside, above or below the optimal and was selected based on availability.

6.3 Communication Between Platforms

In order to keep both players in sync with one another, a remote server was developed to handle all communications between the platforms. Each platform selects a reaction and processes the information through image analysis and the decision-making algorithm as described previously. The selected move is then sent to the remote server from the platform for processing. All logic for the game, such as updating board movements, is handled by the server. Once an iteration of the game has been completed, the server broadcasts a message to all connected clients detailing who has won the game. The players then adjust their strategies accordingly.

The reasoning behind developing a remote server system for this task was a separation of concerns. By separating the game logic from the platforms, as opposed to each platform having its own representation of the game, we minimize the risk of each platform falling out of sync with one another leading to inaccurate results. We also prevent potential race conditions with platforms attempting to access a single file at the same time. A single server with file access eliminates this risk. The design of the server allows for multiple concurrent connections and data processing which opens the possibility of increasing the number networked platforms working towards a common goal.

6.4 Strategy

Both players begin the first game in the sequence by selecting reactions from an identical chemical space (**Supplementary Figure 24 center**). Once the loser of the first game has been established, that player is allowed to access a new strategy/expanded grid (**Supplementary Figure 24 right**) whilst the winner continues with the original. Each strategy/chemical space consists of 9 grids small grids of two aniline derivatives labelled A, B and C (**Supplementary Figure 24 left**). The change of the reaction space from the original to the expanded is achieved by adding two extra values of reagent volume to each of these 9 smaller grids.

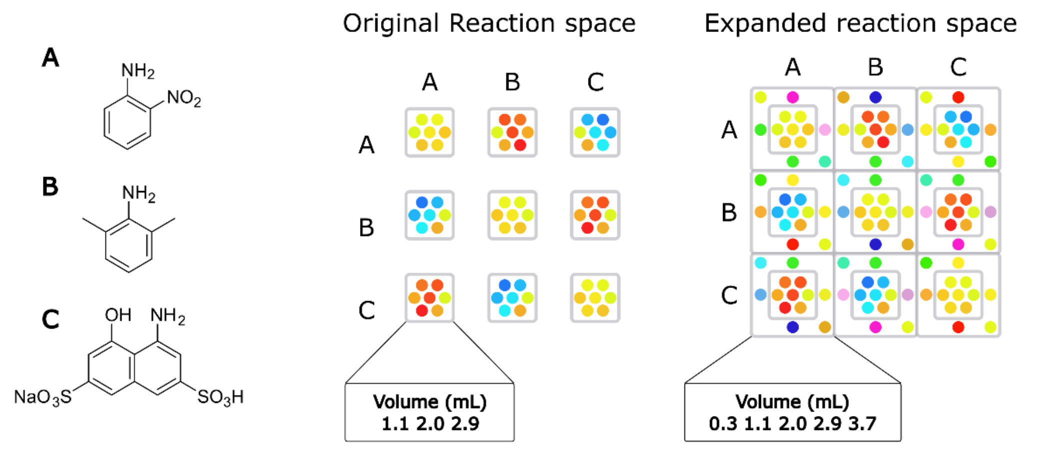

Supplementary Figure 24: Reaction grid layout for game strategies. Aniline derivatives (left) A- o-nitro-aniline, B- 2-6-dimethyl-aniline, C- 4-amino-5-hydroxy-2, 7 napthalenedisulfonate hydrate. Shared original reaction space (Centre), Expanded reaction space (right).

Given that the game sequence proceeds one platform after another the original reaction space restricts the total reaction number to 81 for each player (9 grids of 3x3 reagent volumes). A typical game sequence can consist of between 2-5 completed games. Seen below in **Supplementary Figure 25** is a game sequence showing 4 complete games (5th game was incomplete) in which the losing strategy was adopted by player 2 after game 1. Against the logical expectation the losing strategy, whilst

allowing player 2 to win game 2, did not result in many new unique discoveries. However, when player 1 adopted the losing strategy after game 2, its unique discovery count increased significantly, but did not result in a victory for the remained of the total game sequence. This can be explained simply by the fact a game is still based on probability and a new advantageous strategy will work most, but not all of the time.

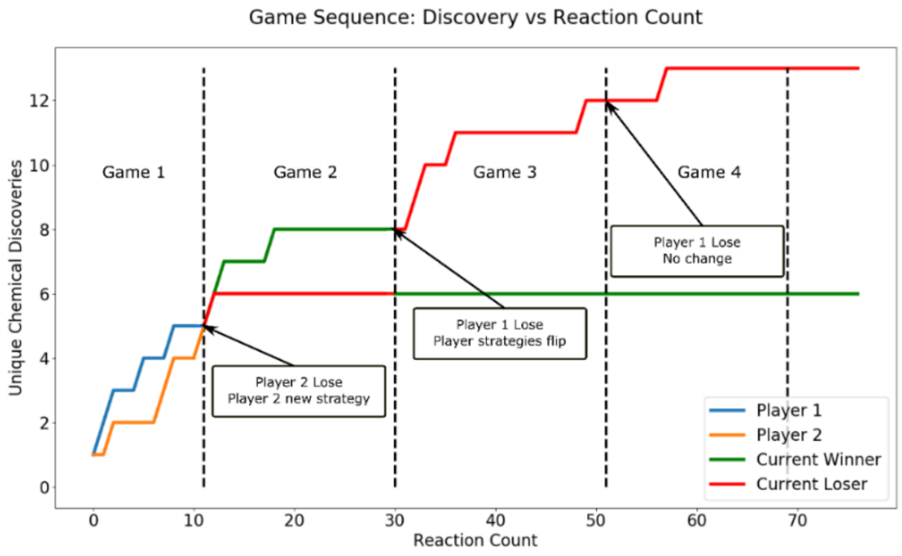

Supplementary Figure 25: **Example game series**. A 4 game sequence showing adoption of a new strategy allows, over time, for an increased number chemical discoveries.
